# Pathologically catalyzed physical coating restores the intestinal barrier for inflammatory bowel disease therapy

**DOI:** 10.1186/s12951-023-02227-0

**Published:** 2023-11-24

**Authors:** Yuge Zhao, Ruiqing He, Jie Zang, Weimin Yin, Runping Su, Wei Xiong, Weihua Xu, Jiaxin Zhang, Yiqiong Liu, Tianbin Ren, Yongzhuo Huang, Yongyong Li

**Affiliations:** 1 Shanghai Tenth People’s Hospital, The Institute for Biomedical Engineering & Nano Science (iNANO), School of Medicine, Tongji University, Shanghai, 200092 China; 2 State Key Laboratory of Drug Research, Shanghai Institute of Materia Medica, Chinese Academy of Sciences, Shanghai, 201203 China

**Keywords:** Polydopamine coating, Pathological catalysis, Intestinal barrier, Inflammatory bowel Disease

## Abstract

Intestinal epithelia impairment of inflammatory bowel disease (IBD) leads to the leakage of bacteria and antigens and the consequent persistent immune imbalance. Restoring the epithelial barrier is a promising therapeutic target but lacks effective and safe clinical interventions. By identifying the catalase (CAT) presence in the IBD pathological environment, we herein develop a CAT-catalyzed pathologically coating on the damaged epithelial barrier to inhibit intestinal leakage for IBD therapy. With the codelivery of CaO_2_ (a CAT substrate) and dopamine, the nanosystem can enable CAT-catalyzed oxygen (O_2_) production and in-situ polymerization of dopamine and then yield a thin and integrative polydopamine (PDA) coating on the intestinal barrier due to the highly adhesive property of PDA. In vivo study demonstrates that PDA coating provides not only a protective barrier by restricting intestinal leakage but also a favorable anti-inflammation effect. Beyond drug management, this work provides a physical repair strategy via catalyzed coating for IBD therapy.

**Supplementary Information:**

The online version contains supplementary material available at 10.1186/s12951-023-02227-0.

## Introduction

The intestinal epithelia serve as a protective barrier against physical and chemical damage, which can sense immune signals and maintain homeostasis [1, 2]. However, inflammatory bowel disease (IBD), a chronic and incurable inflammatory disease [3, 4], is associated with structural damage and dysfunction of the intestinal epithelial barrier [2, 5]. The impaired barrier results in the leakage of bacteria and antigens from the lumen into the underlying layer, thus eliciting persistent inflammation [6, 7]. Therefore, restoring the epithelial barrier has been recently emphasized as a promising therapeutic target for IBD therapy [8–11]. Currently, only a few drugs have been developed for barrier repair (e.g., IL-22, R-Spondin1, and EGF) in clinical and preclinical studies [8, 12–15], based on the principle of promoting epithelial cell proliferation via regulating the related signaling pathways. However, the benefits of these modalities are not only moderate but also at the expense of risking tumorigenesis [8, 16]. Here, we propose an in-situ physical barrier constructed on the intestinal epithelium to inhibit leakage and promote intestinal self-repair as a novel strategy.

Polydopamine (PDA) has been found with outstanding adhesive property and intrinsic biocompatibility, which has a similar molecular structure to the 3,4-dihydroxy-L-phenylalanine (DOPA) in mussels. PDA can act as a coating material on different material surfaces, playing a role in physical isolation, protection, and repair [17–21]. In addition, PDA also has anti-inflammatory effects with high scavenging efficiency of reactive oxygen species (ROS) [22–24]. On this basis, we creatively proposed the strategy of in-situ forming PDA coating on the intestinal epithelium in the IBD pathological microenvironment. However, the oxidation environment is the critical point for the formation of PDA coating, and the anaerobic intestinal environment is the obstacle [25, 26]. By identifying an upregulation of catalase (CAT) in IBD (Figure S1), “catalytic medicine” inspired us to deliver CAT substrates to produce oxygen, ultimately forming pathologically catalyzed PDA physical coatings, which are anti-inflammatory and biocompatible.

**Scheme 1:**
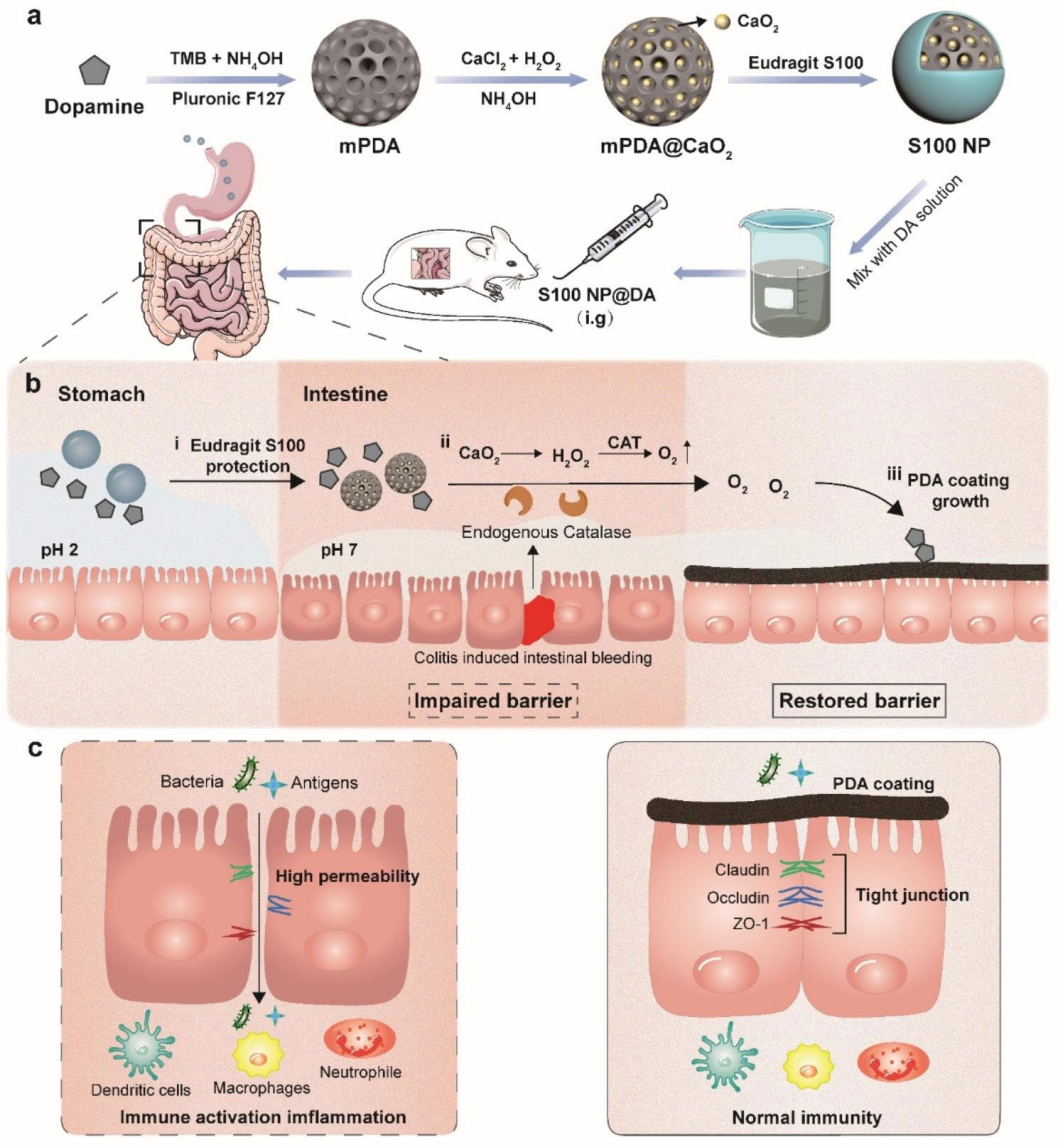
Schematic illustration of S100 NP preparation and the in-situ growth mechanism of the catalase (CAT)-catalyzed polydopamine (PDA) coating to restore intestinal epithelia. (**a**) S100 NP synthesis and intragastric (i.g.) administration with dopamine (DA) solution. (**b**) Behavior in the gastrointestinal tract: (i) the Eudruagit S100 layer protects the S100 NP from premature release in the acidic environment (pH 2) but de-shells in the intestine due to pH change; (ii) calcium peroxide (CaO_2_) exposes and produces oxygen (O_2_) catalyzed by CAT; (iii) DA is oxidized into PDA coating on the diseased epithelia. (**c**) The PDA coating prevents inflammation caused by bacterial and antigen leakage and protects the tight junctions between epithelia

Herein, we developed a strategy of pathologically catalyzed coating that combines “catalytic medicine” and “barrier repair” to treat IBD. As shown in Scheme 1a, calcium peroxide (CaO_2_) is encapsulated into the mesoporous polydopamine (mPDA) nanostructure through a biomineralization approach to form mPDA@CaO_2_. The mPDA@CaO_2_ is then coated by the Eudragit S100 layer (S100 NP), and mixed with dopamine (DA) for oral delivery. The Eudragit S100 layer is a pharmaceutical enteric-coated to protect the nanoparticles from destruction in the acidic environment (pH 2). However, once Eudragit S100 is dissolved in the intestine (pH 7), the loaded CaO_2_ will turn into H_2_O_2_ and serve as an O_2_ source (CaO_2_→H_2_O_2_→O_2_) catalyzed by CAT that is overexpressed in the pathological site. The generated O_2_ triggers the in-situ polymerization of DA into mussels-like adhesive PDA on the pathological intestinal wall (Scheme 1b). The thus-formed PDA coating thereby can provide physical protection to the impaired epithelium barrier (Scheme 1c). This delivery and therapeutic strategy are site-specific in the pathological site by a cascade process including CaO_2_ exposure, H_2_O_2_ generation, CAT-catalyzed O_2_ production, and DA polymerization.

## Results

### CAT-catalyzed formation of intestinal PDA coating

**Fig. 1:**
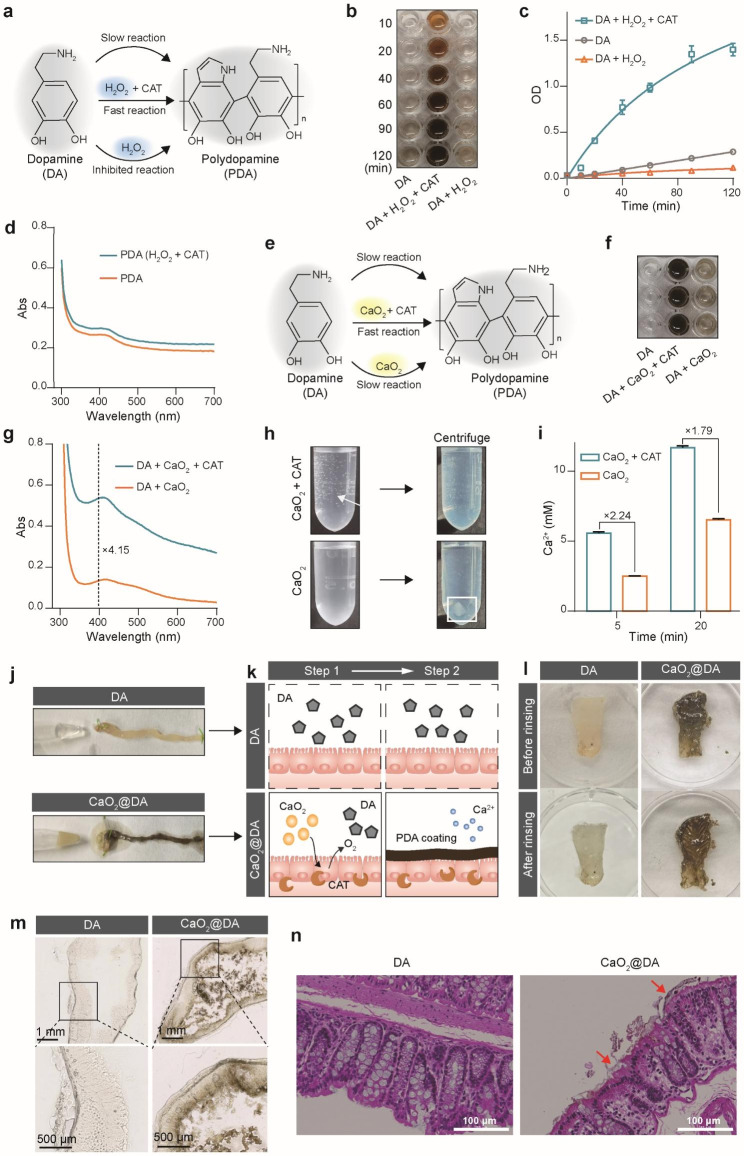
Ex vivo study of CAT-catalyzed PDA growth on the colon tissue. (**a**) Schematic illustration of DA polymerization under different conditions (with H_2_O_2_ and CAT, or H_2_O_2_ alone). (**b**) Visual results of DA polymerization under conditions shown in (**a**) at various time points (DA is colorless and PDA is dark brown). (**c**) OD value of the samples in (**b**) at 700 nm (*n* = 3). (**d**) UV-vis spectra of PDA standard and PDA with CAT catalysis. (**e**) Schematic illustration of DA polymerization under different conditions (with CaO_2_ and CAT, or CaO_2_ alone). (**f**) Visual results of DA polymerization under conditions shown in (**e**). (**g**) UV-vis spectra of PDA under conditions in (**e**). (**h**) Images showing the reaction of CaO_2_ and CAT, or CaO_2_ alone (Left column, the O_2_ is indicated by a white arrow). Image showing the centrifuged solution (Right column, the aggregated CaO_2_ is indicated by the white box). (**i**) [Ca^2+^] in the supernatant after centrifugation of solution in (**h**) (*n* = 3). (**j**) The colon was incubated with DA and CaO_2_@DA ex vivo. (**k**) Schematic illustration of the PDA coating on the epithelium ex vivo. (**l**) Images showing the PDA coating on the intestine. (**m**) Microscopic analysis of the PDA coating on the surface of the epithelium. (**n**) The histological examination (H&E staining) of the colon in DA and DA@CaO_2_ groups (The PDA coating is shown by the red arrows). Results were expressed as mean ± SD.

Generally, the polymerization of DA to form PDA requires an oxidative environment [25]. Considering the hypoxic environment of the intestine [26, 27], we first assessed the feasibility of CAT-catalyzed polymerization of DA in the presence of H_2_O_2_. As shown in Fig. 1a, the polymerization of DA was monitored under different conditions (DA, DA + H_2_O_2_, and DA + H_2_O_2_ + CAT). Without H_2_O_2_ and CAT, the DA (10 mg/mL, 200 µL) was almost unchanged within 2 h, indicating minimal PDA formation. However, in the presence of a trace amount of H_2_O_2_ (3%, 4 µL) and CAT (1 mg/mL, 5 µL), the DA instantly turned dark brown, indicating the rapid PDA formation (Fig. 1b, c). Moreover, H_2_O_2_ alone without the CAT inhibited the polymerization of DA (Fig. 1c), which may be due to the reducibility of H_2_O_2_ inhibits the oxidation of DA. The DA + CAT group also showed no change which further proved the crucial role of H_2_O_2_ (Figure S2). The appearance of 400 nm absorbance, characteristic of PDA in UV-vis spectroscopy, showed the successful PDA formation in the presence of CAT and H_2_O_2_ (Fig. 1d). These results demonstrated that the CAT catalysis of H_2_O_2_ into O_2_ can provide an oxidative environment for DA polymerization.

CaO_2_ can generate H_2_O_2_ (CaO_2_ + H^+^ → Ca^2+^ + H_2_O_2_), and thus solve the problem that H_2_O_2_ is difficult to deliver [28–34]. As shown in Fig. 1e, the polymerization of DA under different conditions (DA, DA + CaO_2_, and DA + CaO_2_ + CAT) showed that polymerization of DA could not be effectively triggered by only adding CaO_2_ (Fig. 1f). Whereas the polymerization of DA was significantly increased in the DA + CaO_2_ + CAT group, which was 4.15 times higher than the DA + CaO_2_ group (Fig. 1g). The increased DA polymerization rate correlated with the up-regulation of CaO_2_ (Figure S3) in the presence of CAT but showed little increase without CAT (Figure S4). It indicated that CAT was critical for the polymerization of DA as it converts H_2_O_2_ generated from CaO_2_ into O_2_. The released O_2_ was visible when adding CAT into the CaO_2_ aqueous dispersion, and no CaO_2_ was left after the reaction (Fig. 1h). Furthermore, [Ca^2+^] in the supernatant with CAT was significantly higher than that without CAT (Fig. 1i). The results suggested that CaO_2_ can be an efficient substrate for O_2_ generation in the pathological intestine. Notably, all the ultimate products (Ca^2+^, H_2_O, O_2_) from this bioreactor are biologically benign.

CAT is an enzyme that mainly presents in mammalian cells, especially in the IBD intestine [35]. The endogenous CAT in the intestinal tract was detected by benzidine and H_2_O_2_ reagent and the highest level of CAT was found in the colon (Figure S5). Therefore, both ends of the colon were ligated to create an oxygen-free environment and then incubated with DA and CaO_2_@DA to investigate in-situ PDA formation on the intestine surface, respectively (Fig. 1j). As shown in Fig. 1k, from step 1 to step 2, the DA showed no change in the colon, while the CaO_2_@DA rapidly turned dark brown, suggesting rapid PDA formation. This proves that CAT promotes the production of O_2_ in the presence of CaO_2_, which in turn promotes the formation of PDA (Fig. 1k). The intestine is dissected along the long axis, and the PDA coating was observed in the CaO_2_@DA group (Fig. 1l) and it firmly remained after rinsing (Fig. 1l). Frozen sections showed the microscopic morphology of the PDA coating, which adhered uniformly to the surface of the intestine (Fig. 1m). The histological details of the colon were examined by H&E staining (Fig. 1n), and the PDA coating was visible in the CaO_2_@DA group (red arrow). Moreover, to investigate the effect of ligation on colon homeostasis, the HIF-1α and ROS were investigated. The results showed that there was no significant difference in HIF-1α and ROS between the control and ligated groups (Figure S6, S7). Therefore, it is feasible to deliver CaO_2_@DA to form PDA coating on the intestine for in vivo applications.

### Synthesis and functional study of CaO_2_ delivery system

**Fig. 2:**
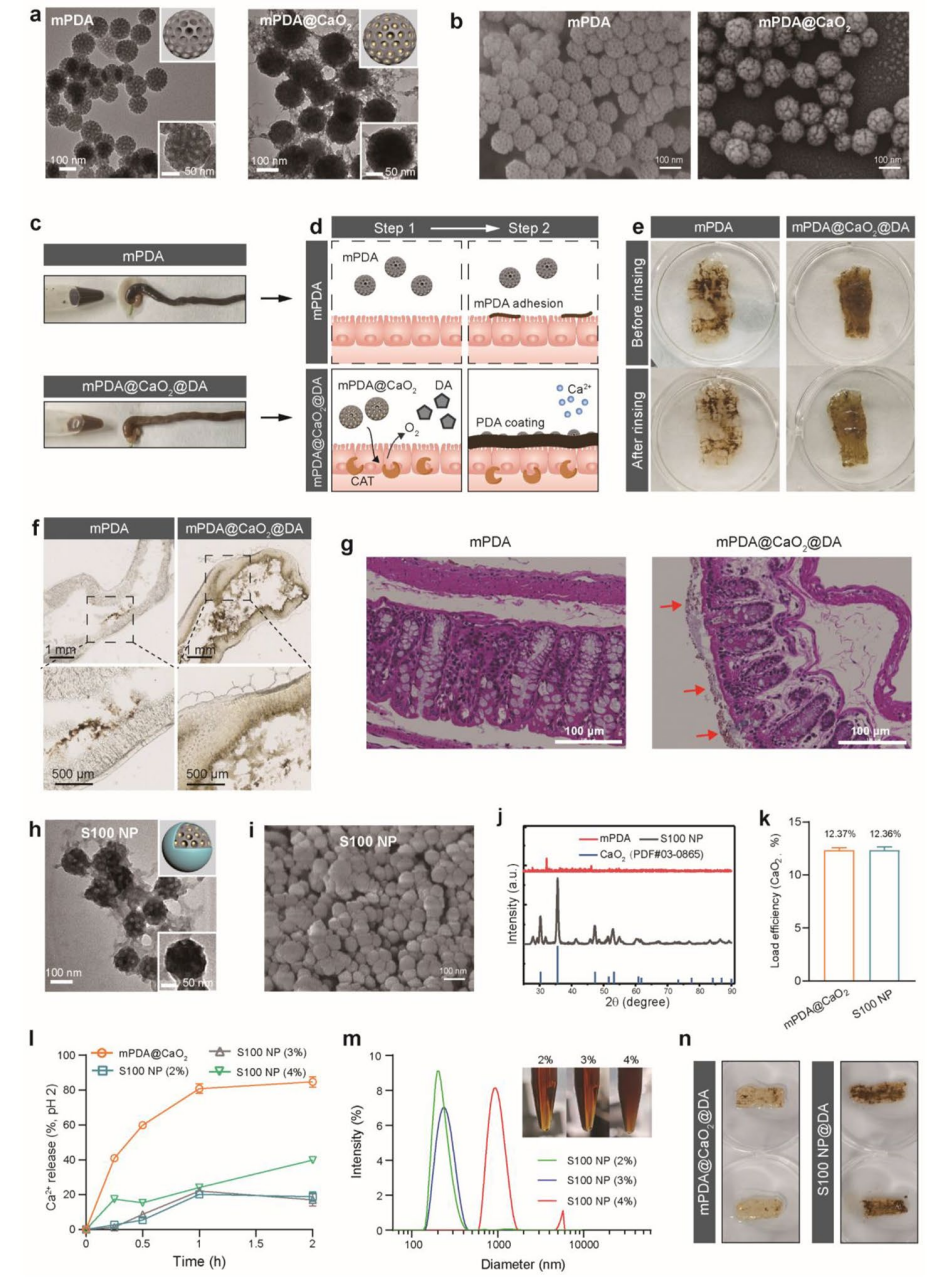
Preparation and functional study of mPDA@CaO_2_ and S100 NP. TEM (**a**) and SEM images (**b**) of mPDA and mPDA@CaO_2_. (**c**) The colon was incubated with mPDA@CaO_2_@DA and mPDA ex vivo. (**d**) Schematic illustration of the growth of PDA coating on the epithelium by mPDA@CaO_2_@DA. (**e**) Images showing the PDA coating on the intestine. (**f**) Microscopic analysis of the PDA coating on the intestine. (**g**) H&E staining of the colon in mPDA and mPDA@CaO_2_@DA groups (The PDA coating is shown by the red arrows). TEM (**h**) and SEM image (**i**) of S100 NP. (**j**) XRD of mPDA, S100 NP, and CaO_2_(PDF#03-0865) (**k**) Loading efficiency of CaO_2_ on mPDA@CaO_2_ and S100 NP (n = 3). (**l**) Ca^2+^ release of mPDA@CaO_2_ and S100 NP in the simulated gastric acid (pH 2) (*n* = 3). (**m**) The diameter of S100 NP with different concentrations of Eudragit S100. (**n**) After co-incubation with simulated gastric acid, the formation of PDA coating on the intestine via mPDA@CaO_2_@DA and S100 NP@DA. Results were expressed as mean ± SD.

Nanoparticles have unique physicochemical properties and have been widely used as the oral delivery system for IBD [36–40]. The mesoporous polydopamine (mPDA), known for its unique metal-chelate property [41], can afford CaO_2_ growth in its mesoporous structure. The TEM and SEM images displayed that the mesoporous structure of mPDA disappears after the growth of CaO_2_ (Fig. 2a, b). The in-situ PDA formation on the tissue surface was investigated by ligation of both ends of the colon and incubation with mPDA@CaO_2_@DA solution (Fig. 2c). As shown in Fig. 2d, from step 1 to step 2, mPDA only partially adhered to the colon, while mPDA@CaO_2_@DA formed a uniform PDA coating. After dissecting the intestines, the PDA coating could remain intact after rinsing (Fig. 2e). The PDA coating on the surface of the intestine can be observed by freezing sections (Fig. 2f). The H&E staining displayed histological details of the colon in mPDA and mPDA@CaO_2_@DA groups (Fig. 2g), and the PDA coating was visible in the mPDA@CaO_2_@DA group (red arrows). In addition, mPDA@DA was unable to form a complete PDA coating on the intestine (Figure S8), which proved that the O_2_ supply of CaO_2_ played an important role in the formation of PDA coating.

The Eudragit S100, a clinic pH-dependent enteric-soluble coating material [42–44], was used as the protective layer of mPDA@CaO_2_ to avoid decomposing by stomach acid. Through coordination of CaO_2_ with carboxyl groups, S100 was wrapped onto the surface of mPDA@CaO_2_ to form S100 NP (Fig. 2h, i), showing improved dispersion in solution (Figure S9). The particle size of mPDA, mPDA@CaO_2_, and S100 NP was all around 150–200 nm with a narrow PDI of 0.1–0.2 (Figure S10a). The negatively charged Eudragit S100 significantly reduced the zeta potential of S100 NP (Figure S10b). The representative diffraction peak in S100 NP matched well with the standard CaO_2_ powder diffraction peak (PDF#03-0865) (Fig. 2j). CaO_2_ can be well tolerated for its degradation capacity into biologically benign elements in the gastrointestinal tract, in consideration of only the 12.3% loading efficiency in mPDA@CaO_2_ and S100 NP (Fig. 2k; Figure S11).

In addition, the protection of Eudragit S100 coating for mPDA@CaO_2_ was investigated by monitoring the Ca^2+^ release in a simulated stomach acid environment. It was found that mPDA@CaO_2_ precipitated significantly in the dialysis bag, while the mPDA@CaO_2_ encapsulated by various concentrations of Eudragit S100 maintained their good dispersity (Figure S12a). About 80% of the Ca^2+^ was released from mPDA@CaO_2_ within 1 h. In contrast, with the Eudragit S100 coating, the Ca^2+^ release only reached 20% in 1 h, which proved most of the CaO_2_ against the acid environment. After 2 h, the Ca^2+^ release of S100 NP (4%) reached 40%, and the release of Ca^2+^ in S100 NP (2%) and S100 NP (3%) remained at 20% (Fig. 2l; Figure S12b). The significantly increased particle size and PDI of S100 NP (4%) may be the cause of the poor release kinetics of Ca^2+^, thus, S100 NP (2%) was selected for further study (Fig. 2m). Furthermore, after pretreating in an acidic environment, the formation of PDA coating can be achieved by S100 NP@DA, while mPDA@CaO_2_@DA could no longer form PDA coating (Fig. 2n).

### The Biocompatibility and anti-inflammatory effect of S100 NP on Caco2 cells

**Fig. 3:**
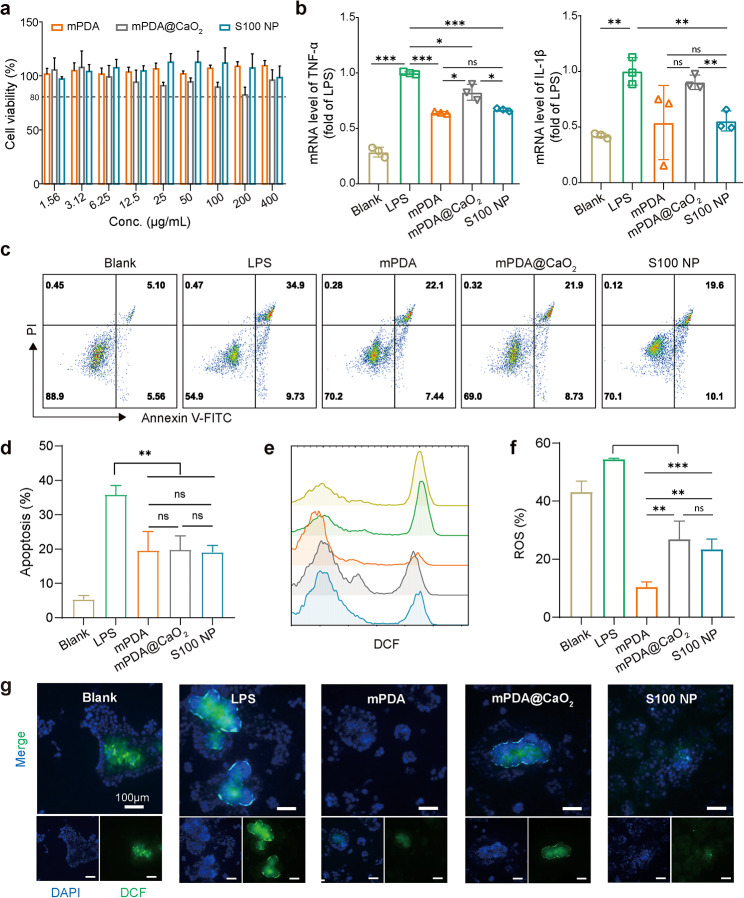
The biocompatibility and anti-inflammatory effect of S100 NP on Caco2. (**a**) The biocompatibility of nanoparticles for Caco2 (*n* = 3). (**b**) The mRNA level of pro-inflammatory cytokines (TNF-α, IL-1β) in the LPS-induced Caco2 (*n* = 3). The flow cytometry (**c**) and quantitative results of apoptosis (**d**) of LPS-induced Caco2 (*n* = 3). (**e**) The flow cytometry analysis of ROS level (Blank, LPS, mPDA, mPDA@CaO_2_, and S100 NP, respectively). (**f**) Quantitative results of ROS level of LPS-induced Caco2 (*n* = 3). (**g**) Fluorescence of ROS in Caco2 (Scale bar: 100 μm). Results were expressed as mean ± SD. *p < 0.05, **p < 0.01, ***p < 0.001; ns represented not significant

To explore the biocompatibility and anti-inflammatory effect of S100 NP for further application, an intestinal epithelial cell line (Caco2) was induced by lipopolysaccharide (LPS) to establish an in vitro inflammatory model [45]. First, the biosafety study of the nanoparticles showed that mPDA, mPDA@CaO_2_, and S100 NP had an unnoticeable toxic effect on Caco2 and the cell viability remained above 80% which met the experimental requirements (Fig. 3a). LPS can stimulate Caco2 raising the inflammatory cytokines [46]. The production of cytokines in LPS-stimulated Caco2 was examined via q-PCR, and the results showed that the mRNA levels of TNF-α and IL-1β were significantly decreased via treatment with nanoparticles (Fig. 3b). Although there was a little rise of these cytokines in the mPDA@CaO_2_ group, it was probably due to direct exposure to CaO_2_. A high concentration of LPS (> 50 µg/mL) decreased the survival rate of Caco2 (Figure S13) and up-regulated the apoptosis of Caco2 (Fig. 3d), which caused the epithelial barrier damage [47]. After co-incubation with nanoparticles, apoptosis of Caco2 was significantly decreased (Fig. 3c, d). As ROS can trigger inflammation [48], the ability of nanoparticles to eliminate the LPS-induced ROS generation in Caco2 was detected by flow cytometry and fluorescence imaging. mPDA significantly decreased ROS compared to LPS-induced Caco2 (about 5.2 fold). Although the mPDA@CaO_2_ and S100 NP groups increased ROS which may be due to the presence of CaO_2_, they still reduced ROS by about 1.0–1.3 fold (Fig. 3e, f). The fluorescence microscopy images also showed that the green fluorescence intensity of the intracellular ROS was significantly decreased (Fig. 3g), which was consistent with flow cytometry. These results indicated that S100 NPs had good biocompatibility with the Caco2, as well as managed to reduce LPS-induced inflammation, apoptosis, and scavenge ROS. We also investigated the protective effect of PDA coating on the tight junction protein (Occludin, ZO-1, and Claudin-1) of Caco2. The results showed that the expression of tight junction protein of Caco2 in mPDA@CaO@DA group was significantly higher than that in the LPS group (Figure S14). This suggests that in situ PDA coating in vivo may play a therapeutic role by protecting tight junction proteins.

### Pathologically catalyzed PDA growth in vivo

**Fig. 4:**
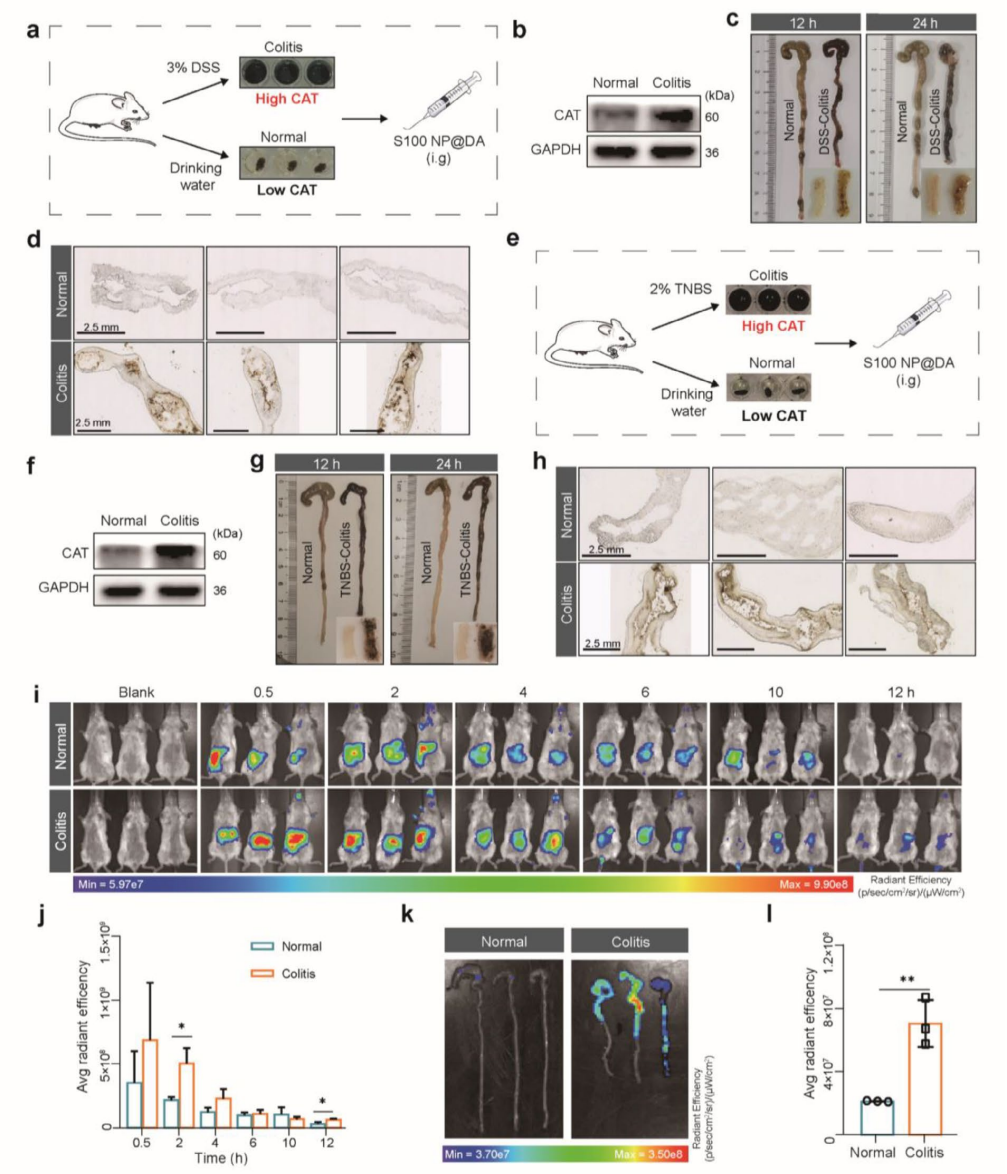
In vivo growth of the pathologically catalyzed PDA coating. (**a**) Schematic illustration of DSS-induced colitis. S100 NP@DA was administered by i.g. (**b**) CAT in the intestine of normal mice and DSS-induced colitis mice detected by western blot. (**c**) Formation of PDA coating on the colon in normal and DSS-colitis mice in vivo. (**d**) Microscopic analysis of the PDA coating on the surface of the colon (Scale bar: 2.5 mm). (**e**) Schematic illustration of TNBS-induced colitis. S100 NP@DA was administered by i.g. (**f**) CAT in the intestine of normal mice and TNBS-induced colitis mice detected by western blot. (**g**) Formation of PDA coating on the colon in normal and TNBS-colitis mice in vivo. (**h**) Microscopic analysis of the PDA coating on the surface of the colon (Scale bar: 2.5 mm). (**i**) In vivo imaging of normal and colitis mice. (**j**) Quantitative analysis of in vivo radiant efficiency (*n* = 3, unit: (p sec^– 1^ cm^– 2^ sr^– 1^) (µW cm^– 2^)^– 1^). (**k**) Ex vivo radiant efficiency of the colon. (**l**) Quantitative analysis of ex vivo colonic radiant efficiency (*n* = 3, unit: (p sec^–1^ cm^–2^ sr^–1^) (µW cm^–2^)^– 1^). Results were expressed as mean ± SD. *p < 0.05, **p < 0.01

To further evaluate the performance of PDA coating in vivo, colitis mice were induced by feeding 3% Dextran sodium sulfate (DSS) solution [27, 49]. As we have validated a large amount of CAT in the intestinal tissue and intestinal tract of colitis mice by western blot and benzidine reagent (Figure, 4b; Figure S1), we hypothesized this intestinal pathological microenvironment of colitis mice is more favorable for CAT-catalyzed PDA coating. To prove this hypothesis, the normal and DSS-induced colitis mice were intragastric (i.g.) with S100 NP@DA solution to characterize in vivo formation of intestinal PDA coating (Fig. 4a). Results showed that the colon of normal mice was negligibly changed, while colitis mice showed a pathological shortening and dark brown colon. After dissecting the colon, the intestinal surface of colitis mice displayed an obvious PDA coating (Fig. 4c), which can remain in the intestine for at least 24 h. The PDA coating on the intestinal surface of colitis mice was further observed in the frozen section (Fig. 4d). However, DSS-induced colitis mice failed to form PDA coating in the intestine after oral administration of DA (Figure S15). In addition, 2,4,6-trinitrobenzenesulfonic acid solution (TNBS)-induced colitis, another classic model, has also verified a high level of CAT in the intestine tissue and intestinal tract by western blot and benzidine reagent (Fig. 4f; Figure S16). Normal and TNBS-induced colitis mice were intragastric (i.g.) with S100 NP@DA solution (Fig. 4e). The results showed that PDA coating was formed on the intestine of TNBS-colitis mice compared with normal mice (Fig. 4g, h), further demonstrating that the formation of PDA coating depends on pathological catalysis.

For a quantitative demonstration of pathologically catalyzed PDA coating in vivo, S100 NP physically loaded near-infrared fluorescence (DIR) was used for in vivo imaging. The fluorescence signal was located predominantly on the abdomen of mice and provided prolonged intestinal retention in colitis mice (Fig. 4i, j), and gradually decreased due to DIR release. The mice were sacrificed for fluorescence detection ex vivo, and no obvious fluorescence was observed in major organs (e.g., heart, liver, spleen, lung, and kidney) (Figure S17). After the colon was removed and rinsed, the colonic fluorescence signal of colitis mice was significantly higher (about 3.3 fold) than that of normal mice (Fig. 4k, l). These results indicate that the IBD pathological microenvironment with high CAT promoted the in-situ growth of PDA coating by the S100 NP@DA system, and realized the pathologically catalyzed physical coating in vivo.

### Therapeutic efficacy of pathologically catalyzed PDA coating against DSS-Colitis

**Fig. 5:**
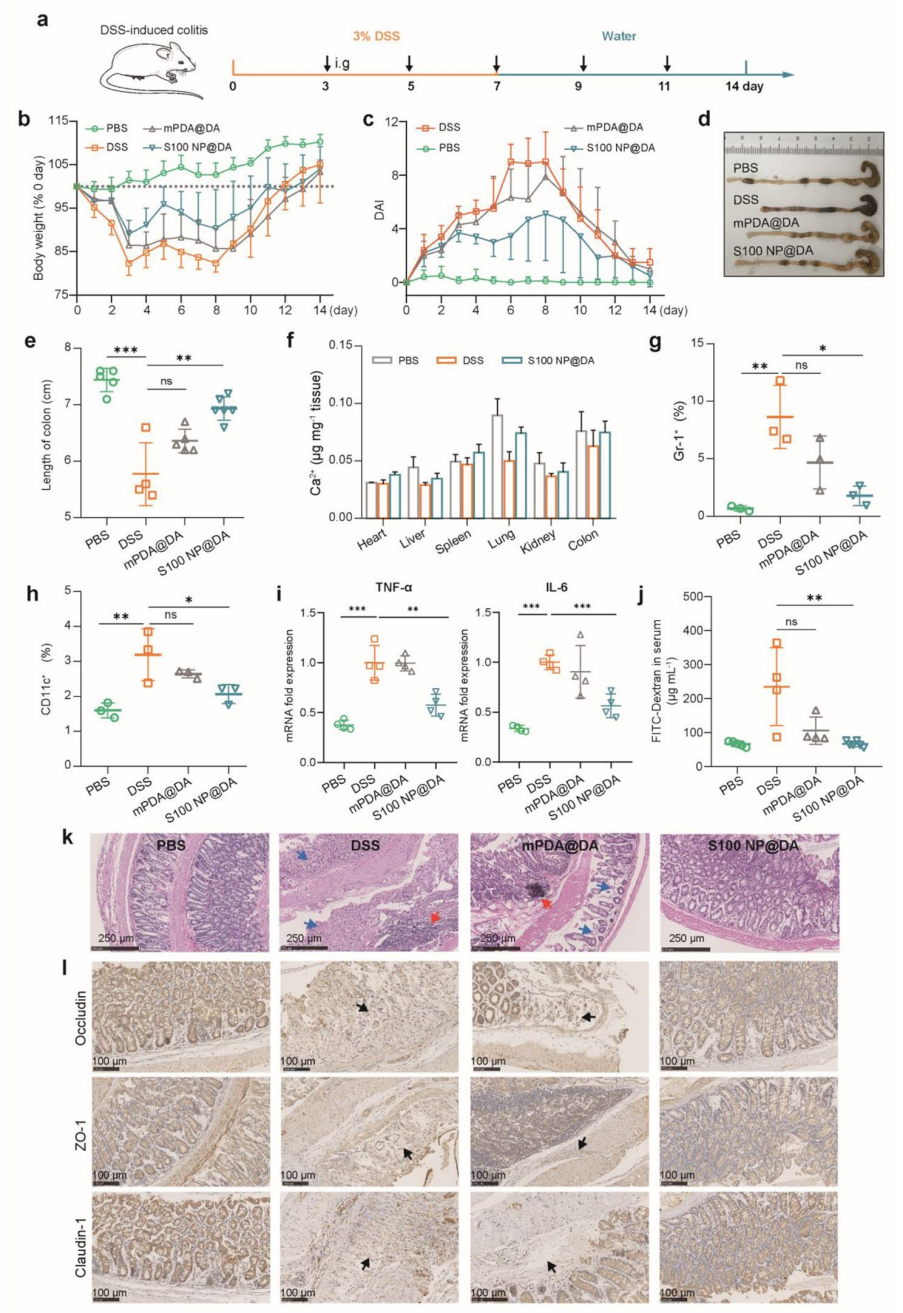
Therapeutic efficacy of pathologically catalyzed PDA coating against DSS-colitis. (**a**) Schematic illustration of colitis induction and treatment. S100 NP@DA was i.g. administered. (**b**) Changes in body weight (*n* = 4–6). (**c**) Disease activity index (DAI) record (*n* = 4–6). (**d**) Image showing the colon tissue of each group. (**e**) Statistical analysis of colon length (*n* = 4–6). (**f**) [Ca^2+^] in major organs and colons (*n* = 3). (**g**) The population of neutrophils (Gr-1^+^) in colon tissue (*n* = 3). (**h**) The population of dendritic cells (CD11c^+^) in colon tissue (*n* = 3). (**i**) The mRNA level of TNF-α and IL-6. (**j**) Intestinal permeability indicated by serum FITC-Dextran (*n* = 4–6). (**k**) The histopathological assessment of the colon with H&E staining. Blue arrow: the loss of the epithelial barrier; Red arrow: the infiltration of immune cells. (**l**) Immunohistochemical staining of Occludin, ZO-1, and Claudin-1 (scale bar: 100 μm). Results were expressed as mean ± SD. *p < 0.05, **p < 0.01, ***p < 0.001; ns represented not significant

Encouraged by the pathologically catalyzed PDA coating growing in the intestinal tract, we explored its therapeutic efficacy against colitis. As shown in Fig. 5a, DSS-induced colitis mice were intragastrically administered with mPDA@DA and S100 NP@DA five times at the same interval (2 days). The DSS-induced colitis mice had significantly lower body weight and increased disease activity index (DAI), while S100 NP@DA reduced weight loss (Fig. 5b; Figure S18) and decreased DAI (Fig. 5c; Figure S19). However, mPDA@DA did not significantly inhibit weight loss and DAI increase in colitis mice. DSS typically caused colonic shortening compared with normal mice (i.e., PBS group), and S100 NP@DA could significantly inhibit colon shortening (Fig. 5d, e). However, the colonic length displayed no significant difference between the mPDA@DA group and the DSS group. The [Ca^2+^] in major organs and serum showed unnoticeable change, demonstrating no excessive accumulation of Ca^2+^ and therefore no additional effect (Fig. 5f; Figure S20). Flow cytometry of intestinal immune cells showed that the proportion of neutrophils (Gr-1^+^) and dendritic cells (CD11c^+^) was significantly increased in the DSS group, while the S100 NP@DA group significantly down-regulated the proportion of neutrophils (Fig. 5g; Figure S21) and dendritic cells (Fig. 5h; Figure S21). The M2 cells which are a benefit to anti-inflammation increased in the S100 NP@DA group (Figure S22). Inflammatory cytokines (such as TNF-α and IL-6) in colon tissue were detected by q-PCR, and the TNF-α and IL-6 were significantly reduced in S100 NP@DA group compared with the DSS group (Fig. 5i). In addition, there was no significant difference in organ coefficients (Figure S23) and no obvious pathological changes in major organs (Figure S24), which indicated the biosafety of the S100 NP@DA. The above results demonstrated that the pathologically catalyzed PDA coating based on the IBD microenvironment displayed a therapeutic effect against colitis.

Colitis is characterized by enhanced intestinal permeability and orally administered FITC-dextran (3,000–5,000 Da) is an indicator for evaluating intestinal permeability. The DSS group had a high level of FITC-Dextran in serum, indicating serious damage to the intestinal epithelial barrier. However, the level of FITC-Dextran in the S100 NP@DA group was close to that in the PBS group (Fig. 5j), suggesting the repair of the intestinal epithelial barrier. Pathological analysis showed that the intestinal epithelial cells of colitic mice were severely damaged, and the epithelial barrier had extensive erosions, accompanied by numerous immune cell infiltration. The S100 NP@DA group significantly reduced epithelial barrier damage as well as immune cell infiltration (Fig. 5k). Furthermore, rising studies indicate that tight junction between epithelial cells significantly depends on tight junction proteins (e.g., Occludin, ZO-1, and Claudin-1) [10, 50, 51]. Immunohistochemical results showed that the S100 NP@DA group could significantly up-regulate the expression of Occludin, ZO-1, and Claudin-1, which have a low level in colitis mice, and the arrows showed Occludin, ZO-1, and Claudin-1 deletion in the DSS and mPDA@DA group (Fig. 5l), further demonstrating the protection and repair of epithelial barrier by pathologically catalyzed PDA coating.

### Further application of pathologically catalyzed PDA coating in TNBS-Colitis

**Fig. 6:**
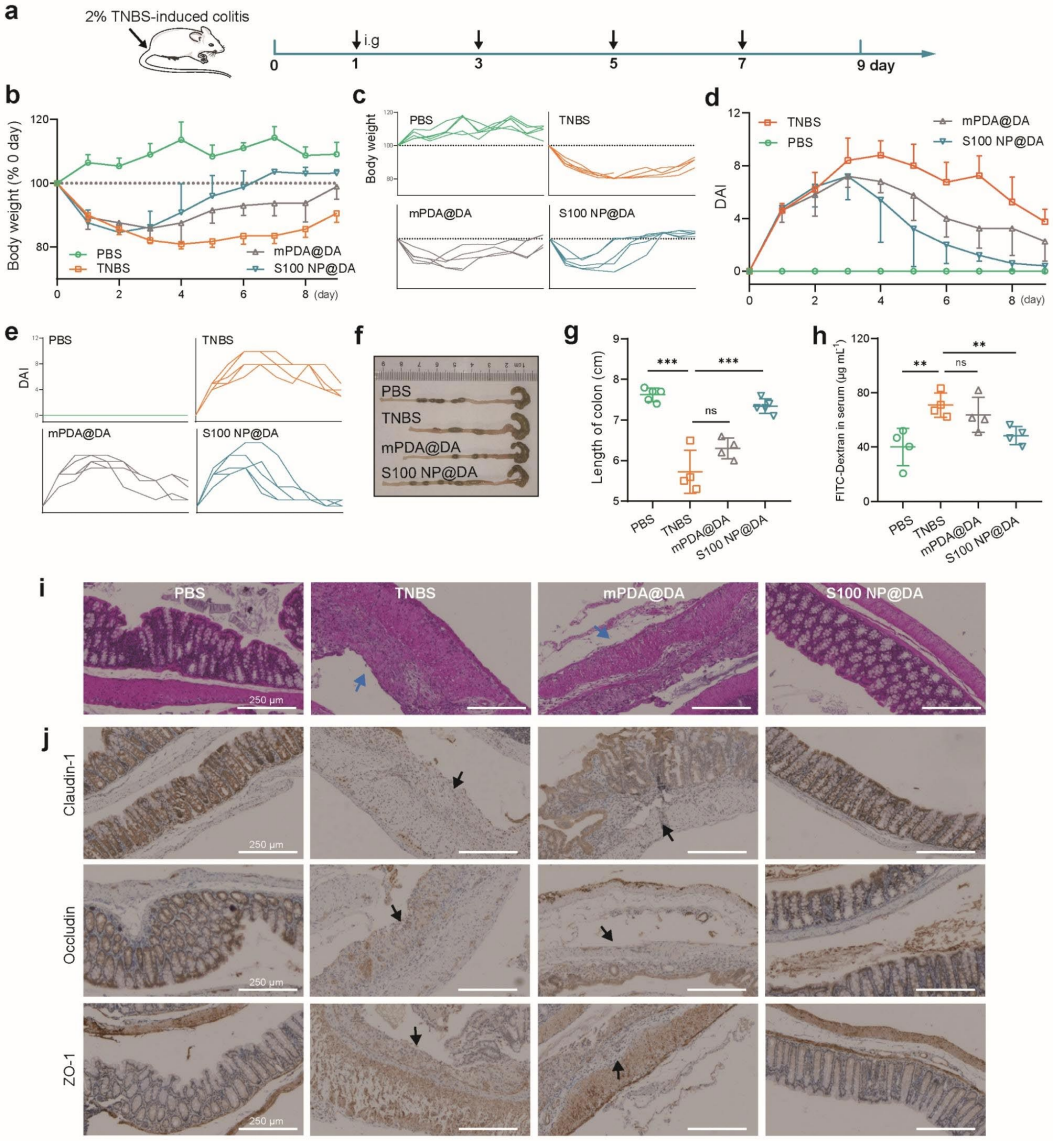
Therapeutic efficacy of pathologically catalyzed PDA coating against TNBS-colitis. (**a**) Schematic illustration of colitis induction and treatment. S100 NP@DA was i.g. administered. (**b**) Changes in body weight (n = 4–5). (**c**) Individual body weight (%) of each group. (**d**) DAI record (n = 4–5). (**e**) Individual DAI of each group. (**f**) Image showing the colon tissue of each group. (**g**) Statistical analysis of colon length (n = 4–5). (**h**) Intestinal permeability indicated by serum FITC-Dextran (n = 4). (**i**) The histopathological assessment of the colon with H&E staining (scale bar: 250 μm). Blue arrow: the loss of the epithelial barrier. (**i**) Immunohistochemical staining of Claudin-1, Occludin, and ZO-1 (scale bar: 250 μm). Results were expressed as mean ± SD. *p < 0.05, **p < 0.01, ***p < 0.001; ns represented not significant

To further investigate the universality of pathologically catalyzed PDA coating in the treatment of colitis, we investigated its therapeutic efficacy in TNBS-induced colitis. As shown in Fig. 6a, mice were injected with 2% TNBS through the anus to induce colitis and intragastrically administered with mPDA@DA and S100NP@DA solution four times at the same interval (2 days). Results showed that TNBS significantly reduced body weight and increased DAI in mice, while S100 NP@DA effectively prevented weight loss (Fig. 6b, c) and DAI increase (Fig. 6d, e). TNBS significantly shortened colon length, while S100 NP@DA significantly inhibited colon shortening, but there was no significant difference between mPDA@DA and TNBS (Fig. 6f, g). Intestinal permeability was verified by oral administration of FITC-dextran (3,000–5,000 Da). The FITC-dextran in the serum of TNBS was significantly increased, while S100 NP@DA could reduce FITC-dextran in serum, indicating decreased intestinal permeability, while mPDA@DA had no significant difference with TNBS (Fig. 6h). Pathological analysis showed that intestinal epithelial in TNBS colitis mice were severely damaged and eroded, and S100 NP@DA restored the integrity of the epithelial barrier (Fig. 6i). In addition, immunohistochemical results showed that the expression of tight junction proteins (Claudin-1, Occludin, and ZO-1) decreased significantly in the TNBS group and recovered in S100 NP@DA, while they remained low expression in the mPDA@DA group (arrows showed Occludin, ZO-1, and Claudin-1 decrease) (Fig. 6j). These results demonstrate the potential universality of the pathologically catalyzed PDA coating and its applicability to the treatment of TNBS colitis.

## Discussion

The intestinal epithelial barrier has been recognized as an important therapeutic target for IBD [8, 11]. The damaged intestinal epithelial barrier not only induces a vicious cycle of inflammation but also results in the occurrence of IBD, a role of provocateur [52]. Traditional IBD drugs (e.g., aminosalicylic acid and glucocorticoids) inhibit immune cell activation or reduce inflammatory cytokine release [53–55], but fail to repair the epithelial barrier [9]. Several agents for epithelial barrier repair have been under development, such as the antibody drug UTTR1147A (NCT03558152). However, these drugs have a long treatment cycle (e.g., UTTR1147A lasted 30 weeks) [13, 56], probably due to the inability to quickly solve intestinal leakage. In this study, we constructed an in-situ physical coating on the intestinal epithelia to timely prevent intestinal leakage. This is crucial to precondition the intestinal self-repair with upregulation of the expression of tight junction proteins including Occludin, ZO-1, and Claudin-1.

To identify a trigger to realize in-situ coating, we found a high level of CAT in the IBD pathological microenvironment. This is due to the damage of the epithelia in IBD, which leads to bleeding in the intestine, resulting in a surge of CAT in the erythrocyte entering the intestinal lumen [57]. As an important antioxidant enzyme, CAT can decompose H_2_O_2_ into water and O_2_ and avoid ROS poisoning in the body [58]. Recently, catalytic medicine has been developing rapidly [59]. Catalytic chemical reactions initiated in microenvironments by nanomaterials with enzyme-like activity, such as single-atom catalysts, show great potential in disease treatment [60–62]. This inspired us to use the catalytic reactions of CAT in the IBD pathological microenvironment to trigger the formation of physical coatings. Therefore, we chose PDA coating for this strategy, because PDA has a mussels-like adhesive property [20, 21], high biocompatibility [18, 19, 63], anti-inflammatory effects [22, 23], and O_2_-dependent polymerization [64]. We first developed the in-situ self-assembly of PDA coating by CAT in the IBD pathological microenvironment, called “pathologically catalyzed PDA coating”, to restore the intestinal epithelial barrier.

This strategy combines the novel concepts of “catalytic medicine” and “pathologically selective coating” to achieve pathologically catalyzed PDA coating using a nanoplatform. Despite the published work realizing the growth of polydopamine onto the intestinal barrier, it employs clinically inviable approaches such as an extreme form of administration, which is pumped through a catheter into the intestine after the removal of gastric fluid and the intestines ligation [18]. Our strategy displayed better clinical translation promise for the reasons: (1) the nanosystem we developed can be orally administrated thus patients can have better compliance; (2) it responds to the pathological environment of IBD to trigger the in-situ growth of polydopamine coating which displayed pathologically selective; (3) PDA is biocompatible and biodegradable, with rising applications in biological interfaces [19, 21, 63–65], such as cell and tissue surfaces; (4) moreover, it may solve the growing shortcomings of traditional drugs via combined administration. Significantly, to our best knowledge, no relevant research has ever been reported for IBD treatment based on a physical barrier-restoring strategy.

## Conclusion

In summary, we developed a pathologically catalyzed PDA coating grown in situ for physical barrier repair in the IBD treatment. The S100 NP@DA system can achieve resistance to gastric acid and selective exposure of CaO_2_ in the intestine. The CAT rapidly decomposes CaO_2_ to produce O_2_, which triggers the growth of PDA coating, finally realizing the “pathologically catalyzed PDA coating”. In colitis mice, PDA coating displayed a promising therapeutic effect and significantly improved intestinal permeability. It also promotes the tight junction protein-mediated repair mechanism. This pathologically catalyzed PDA coating paves a new therapeutic path for IBD.

## Experimental section

### Materials

Dopamine hydrochloride, Pluronic® F-127, Lipopolysaccharide (LPS), and FITC-Dextran (Sigma-Aldrich, Darmstadt, Germany). 1,3,5-Trimethylbenzene (TMB), 28% Ammonium hydroxide solution (NH_3_·H_2_O), Calcium chloride anhydrous (CaCl_2_), Hydrogen peroxide solution (H_2_O_2_), and Catalase (CAT) (Aladdin, Shanghai, China). Benzidine kit (Jiancheng Institute of Biology, Nanjing, China). Calcium Colorimetric Assay Kit, Cell counting kit-8 (CCK8), and ROS detection kit (Beyotime, Shanghai, China). Apoptosis kit, DiR iodide, and 2,4,6-trinitrobenzenesulfonic acid solution (TNBS) (Meilun Biotechnology, Dalian, China). Dextran Sulfate Sodium (DSS), Collagenase IV, RNA extraction reagent, and RNA reverse transcription kit (YEASEN, Shanghai, China). Eudragit S100 (Methacrylic acid and methyl methacrylate copolymer, 1:2) (Evonik Nutrition and Care GmbH, Darmstadt, Germany).

### Cell lines

Human colonic epithelial cells (Caco2) were purchased from the Shanghai Cell Bank of the Chinese Academy of Science (Shanghai, China). Caco2 was cultured in RPMI-1640 medium supplemented with 10% FBS and streptomycin-penicillin (100 U/mL) at 37 °C in a humidified incubator containing 5% CO_2_.

### Animals

BALB/c female mice (about 8 weeks, 18 g) were obtained from Shanghai Laboratory Animal Center, Chinese Academy of Sciences, China. The mice were raised in a specific pathogen-free (SPF) environment. Animal experiments were performed following the Institutional Animal Care and Use Committee (IACUC) guidelines and approved by the Shanghai Institute of Materia Medica (SIMM), Chinese Academy of Sciences, Shanghai, China (Animal Ethics: 2019-01-HYZ-72).

### In Vitro evaluation of CAT-catalyzed PDA polymerization

Dopamine hydrochloride was dissolved in 1 × phosphate-buffered saline (1 × PBS) buffer and added to a 96-well plate (10 mg/mL, 200 µL), then added H_2_O_2_ (3%, 4 µL) and CAT (1 mg/mL, 5 µL). DA group, DA + H_2_O_2_ group, and DA + CAT group were used as control, respectively. The reaction lasted for 120 min, and the OD value at 700 nm was measured at different time points using a plate reader (Multiskan, Thermo Fisher, Waltham, MA, USA). Finally, the PDA solutions were measured by UV-Vis spectroscopy (50 Conc, VARIAN, California, USA).

In CaCl_2_ (500 mM) solution, H_2_O_2_ (30%) and NH_3_·H_2_O (30%) were added and reacted for 30 min, stirred at 500 rpm to produce calcium peroxide (CaO_2_). DA solution was added to a 96-well plate (10 mg/mL, 200 µL), then add CAT (1 mg/mL, 5 µL) and CaO_2_. DA group and CAT-free group were used as control. Finally, the PDA solutions were measured by UV-Vis spectroscopy.

### Detection of CAT expression

The small intestine, cecum, and colon tissues were collected (10 mg respectively), and the tissues were homogenized to obtain tissue lysates. Tissue lysates were diluted 10 times and the CAT in tissue lysates was detected by a benzidine kit. The excreta of normal and colitis mice were collected, and the CAT was also detected by a benzidine kit. The shade of blue indicates the level of CAT.

### Ex vivo formation of PDA coating on tissues

The colon tissues of mice were obtained and rinsed with PBS to remove the contents in the intestinal cavity. The tissues were incubated with DA solution (10 mg/mL), CaO_2_@DA, mPDA (10 mg/mL), mPDA@CaO_2_@DA. 30 min later, the intestinal segment was dissected along the long axis, cleaned gently in PBS, and photographed. The pipette absorbed PBS, flushed the colon, and photographed. The colon tissue was embedded with an embedding agent, frozen and sectioned, and then imaged with a sectioning scanner (NanoZoomer 2.0 HT, Hamamatsu, Japan) to observe the microscopic state of PDA coating. H&E staining and immunohistochemical staining were also performed on colon tissues.

### Preparation and functional study of S100 NP@DA System

DA (50 mg) and Pluronic® F-127 (100 mg) dissolved in 50% ethanol (10 mL) and stirred until transparent. TMB (200 µL) was added drop by drop, the solution turned white. The reaction mixture was stirred for 30 min at room temperature. Then 500 µL NH_3_·H_2_O was added and reacted under the same conditions for 1.5–2 h. The color of the solution turned brown and gradually deepened. The solution was centrifuged at 13,000 rpm for 15 min, the supernatant was discarded, and 50% ethanol was added for re-suspension. Ultrasound was performed for 5–10 min, repeated twice to wash away the excess reactants. Finally, the precipitate was suspended with water to obtain mesoporous polydopamine (mPDA). The CaCl_2_ (50 mM, 100µL) was added to mPDA (1 mg/mL, 1 mL), stir for a while, then added H_2_O_2_ (30%, 100 µL) and NH_3_·H_2_O (100 µL) in turn, stir at room temperature at 500 rpm for 30 min. After centrifugation at 13,000 rpm for 10 min, the supernatant was discarded, washed with PBS, ultrasound was performed for 5–10 min, and then mPDA loaded with CaO_2_ (mPDA@CaO_2_) was obtained. The mPDA@CaO_2_ nanoparticles (1 mg/mL, 1 mL) were stirred slowly, and 0.1 mL Eudragit S100 ethanol solution of different concentrations (2%, 3%, 4%) was slowly added, respectively. Stir at room temperature until the ethanol dried. After centrifugation, S100 NP was obtained by re-suspension with PBS.

The particle size, polydispersity index (PDI), and zeta potential of the nanoparticles were determined by Zeta Size Nanoparticle Analyzer (Malvern Panalytical, Malvern, UK). The nanoparticles were imaged by transmission electron microscopy (TEM) (Talos L120C, FEI, America) and scanning electron microscope (SEM) (Hitachi, Regulus 8100, Japan). The load of CaO_2_ is verified by X-Ray Diffractometer (XRD) (Rigaku, D/max2500, Japan). The load of CaO_2_ was calculated by the standard Ca^2+^ curve.

Ca^2+^ release was measured by the dialysis method. Dialysis bags (MWCO 1000 Da) containing nanoparticles were placed in 30 mL dialysate (pH 2). 0.5 mL dialysate was taken at a preset time point, and 0.5 mL dialysate was supplemented. Ca^2+^ concentration was measured by Calcium Colorimetric Assay Kit.

### Cell viability assay

The Caco2 was planted in a 96-well plate (5000 cells/well) for 24 h and co-incubated with mPDA, mPDA@CaO_2_, and S100 NP. Cell viability was detected by CCK8 after 24 h.

The Caco2 was planted in 96-well plates (10,000 cells/well) for 24 h, and incubated with LPS (100, 50, 25, 12.5, 6.25, 3.12, 1.56, 0.78 µg/mL) for 48 h. Cell viability was detected by CCK8.

### Cytokine analysis by q-PCR

RNA extracted from the Caco2 cells was used to process RNA-to-cDNA transcription using a reverse transcription kit for quantitative PCR according to a standard protocol. The amplification was processed for 1 cycle at 95 °C for 5 min and 40 cycles at 95 °C (10 s), 60 °C (20 s), and 72 °C (20 s).

### Cell apoptosis assay

The Caco2 was cultured in 12-well plates (50,000 cells/well) for 24 h. The LPS-induced (100 µg/mL) Caco2 were incubated with mPDA, mPDA@CaO_2,_ and S100 NP respectively (mPDA 50 µg/mL for each group was the standard) for 12 h. Apoptosis was detected by apoptosis kit and flow cytometry.

### Measurement of intracellular ROS

The Caco2 were plated in 12-well plates (10,000 cells/well) and co-incubated with nanoparticles. LPS was used to induce cells to produce ROS. After 4 h, ROS detection kits were used to test ROS by flow cytometry and fluorescence microscopy, respectively.

### In vivo formation of PDA Coating

Mice with colitis were induced by drinking water containing 3% DSS. Normal mice and colitis mice fasted for 24 h, and S100 NP@DA was given by intragastric administration. The mice were sacrificed and dissected at 12 and 24 h. The colon was collected and the intestinal contents were rinsed. The intestinal tract was dissected along the long axis for photography and the frozen section.

### In vivo imaging of the PDA coating

DIR was loaded on mPDA nanoparticles to prepare near-infrared fluorescence-labeled S100 NP for in vivo imaging. Normal mice and colitis mice fasted for 24 h, and DIR-labeled S100 NP@DA was given by intragastric administration. In vivo imaging was performed at the preset time point via IVIS (Caliper PerkinElmer, Hopkinton, USA). Mice were sacrificed and dissected at 12 h, and the main organs (heart, liver, spleen, lung, and kidney) and colon were taken for ex vivo fluorescence imaging, and the fluorescence intensity was statistically analyzed (Ex: 745 nm, Em: 780 nm).

### Evaluation of Colitis Treatment Efficacy

DSS-induced colitis: mice were fed with 3% DSS drinking water for 7 days and normal drinking water for 7 days respectively to induce colitis. TNBS-induced colitis: mice were fasted for 24 h but with access to drinking water. TNBS (5%, w/v) was diluted with anhydrous ethanol and normal saline to obtain a 2% (w/v) TNBS solution. After anesthesia, TNBS (2%, 100 µL) was injected into the intestine via a catheter (diameter 2.0 mm) inserted into the anus 4 cm proximal with a contact time of 1 min. Mice with colitis were randomized into the untreated group (DSS group) and treatment group (mPDA@DA, S100 NP@DA), and the normal mice group (PBS group) as the control. During this period, treatment was performed by intragastric administration (DA, 10 mg/mL and mPDA, 100 mg/kg), and the mice fasted for 24 h before treatment.

During the treatment, body weight changes, visible stool consistency, and fecal bleeding were assessed daily for determining the disease activity index (DAI) which serves as the summation of the stool consistency index (0–3), fecal bleeding index (0–3), and weight loss index (0–4). After treatments, the colonic length (from the cecum to the rectum) was determined. The main organs (heart, liver, spleen, lung, and kidney) were weighed, the organ coefficients were calculated, and a histological examination was performed. The colon tissue was examined by pathology and immunohistochemistry.

### Analysis of intestinal permeability

After fasting for 24 h, the colitis mice and normal mice were given FITC-Dextran (40 mg/mL in PBS) with a dose of 200 mg/kg via intragastric administration. After 4 h, blood was sampled from the orbit, and the serum concentration of FITC-Dextran was detected (Ex: 488 nm, Em: 525 nm).

### Analysis of Colonic Immune cells

The procedure was modified from a previous report. Colon tissues were dissected, opened longitudinally, and then incubated with the HBSS solution containing DTT and EDTA. The mucus was washed away with cold PBS. Colon tissues were cut into 1 cm pieces for enzymatic digestion (RPMI, 2% FBS, 200 U/mL collagenase type IV) at 37 °C for 1 h. The cell suspension prepared from the digested tissue was incubated with the target antibodies for flow cytometric assay.

### Detection of Ca^2+^ in tissues and serum

The main organs (heart, liver, spleen, lung, kidney) and colon tissues of mice were collected and homogenized to obtain tissue lysate, and serum was extracted after blood sampling from the orbit of mice. Ca^2+^ content in tissues and serum were determined via the Ca^2+^ Colorimetric Assay Kit.

### Statistical analysis

T-test and one-way ANOVA were used for statistical analysis. Data are presented as mean ± standard deviation (SD). N value was 3 if it was not specified. Statistical differences were defined as *p < 0.05, **p < 0.01, and ***p < 0.001; ns means not significant.

### Electronic supplementary material

Below is the link to the electronic supplementary material.

Supplementary Material 1

## Data Availability

Data will be made available on request.
