# Supplementary material for: Pathologically catalyzed physical coating restores the intestinal barrier for inflammatory bowel disease therapy
Source: J Nanobiotechnology. 2023 Nov 24;21:444. doi: 10.1186/s12951-023-02227-0 (PMC10668504; doi:10.1186/s12951-023-02227-0)
Supplement: Supplementary file 1 — Supplementary Material 1 [file 12951_2023_2227_MOESM1_ESM.docx]

**Supporting information**

**Title:**

**Pathologically catalyzed physical coating restores the intestinal barrier for inflammatory bowel disease therapy**

*Yuge Zhao, Ruiqing He, Jie Zang, Weimin Yin, Runping Su, Wei Xiong, Weihua Xu, Jiaxin Zhang, Yiqiong Liu, Tianbin Ren, Yongzhuo Huang,* and Yongyong Li**

Y. Zhao, R. He, J.Zang, W. Yin, R. Su, Y. Liu, T.Ren, Prof. Y. Li

Shanghai Tenth People’s Hospital, The Institute for Biomedical Engineering & Nano Science (iNANO), School of Medicine, Tongji University, Shanghai 200092, China

Email: yongyong_li@tongji.edu.cn

W. Xiong, W. Xu, J. Zhang, Prof. Y. Huang

State Key Laboratory of Drug Research, Shanghai Institute of Materia Medica, Chinese Academy of Sciences, Shanghai 201203, China

Email: yzhuang@simm.ac.cn

**Supplementary Figures**

**
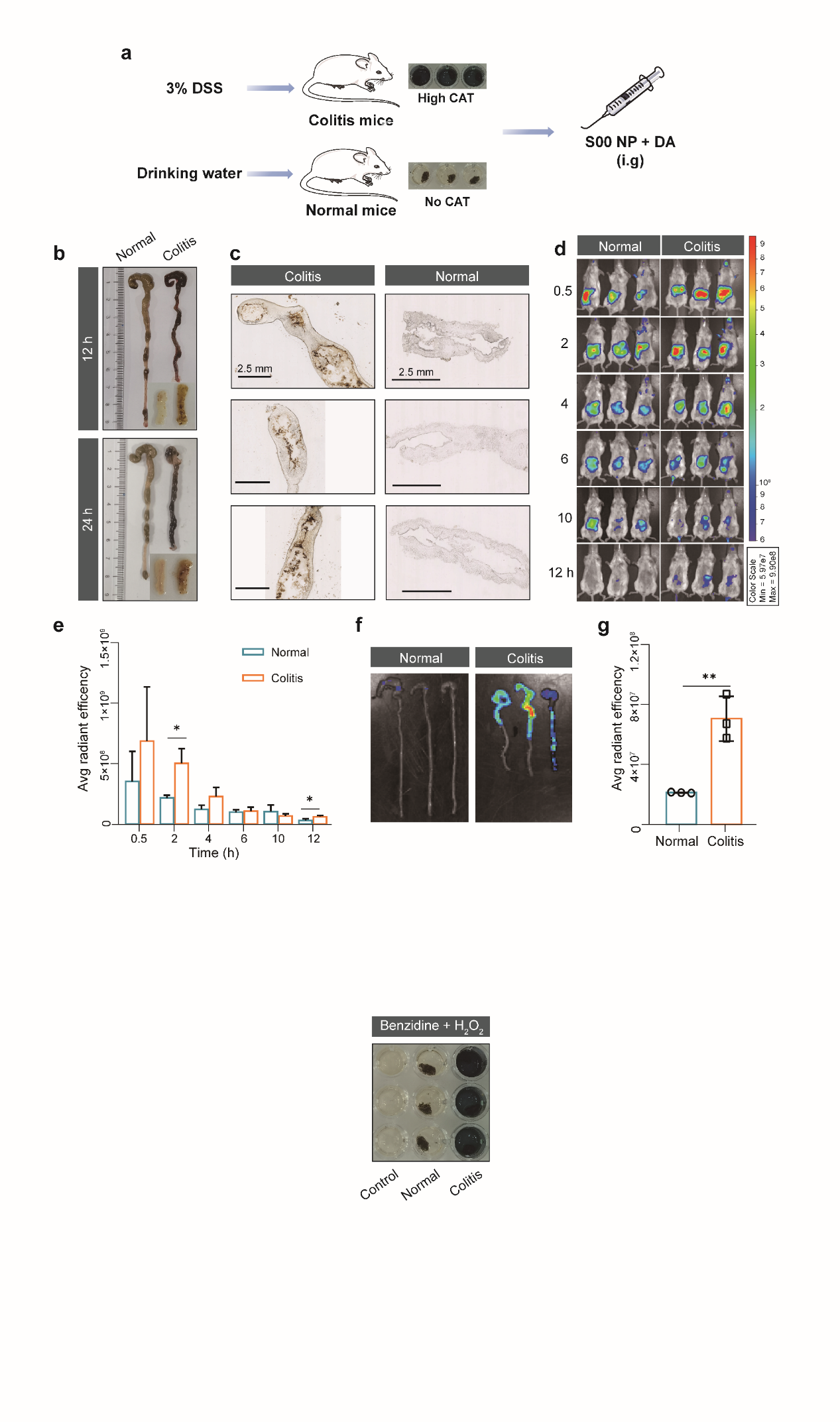
**

**Figure S1**. Expression of CAT in the intestine was detected by Benzidine and H_2_O_2_, and colitis mice have high levels of CAT which turned the solution into dark blue, while the normal mice kept the solution colorless (the blue color of benzidine indicates the level of CAT).


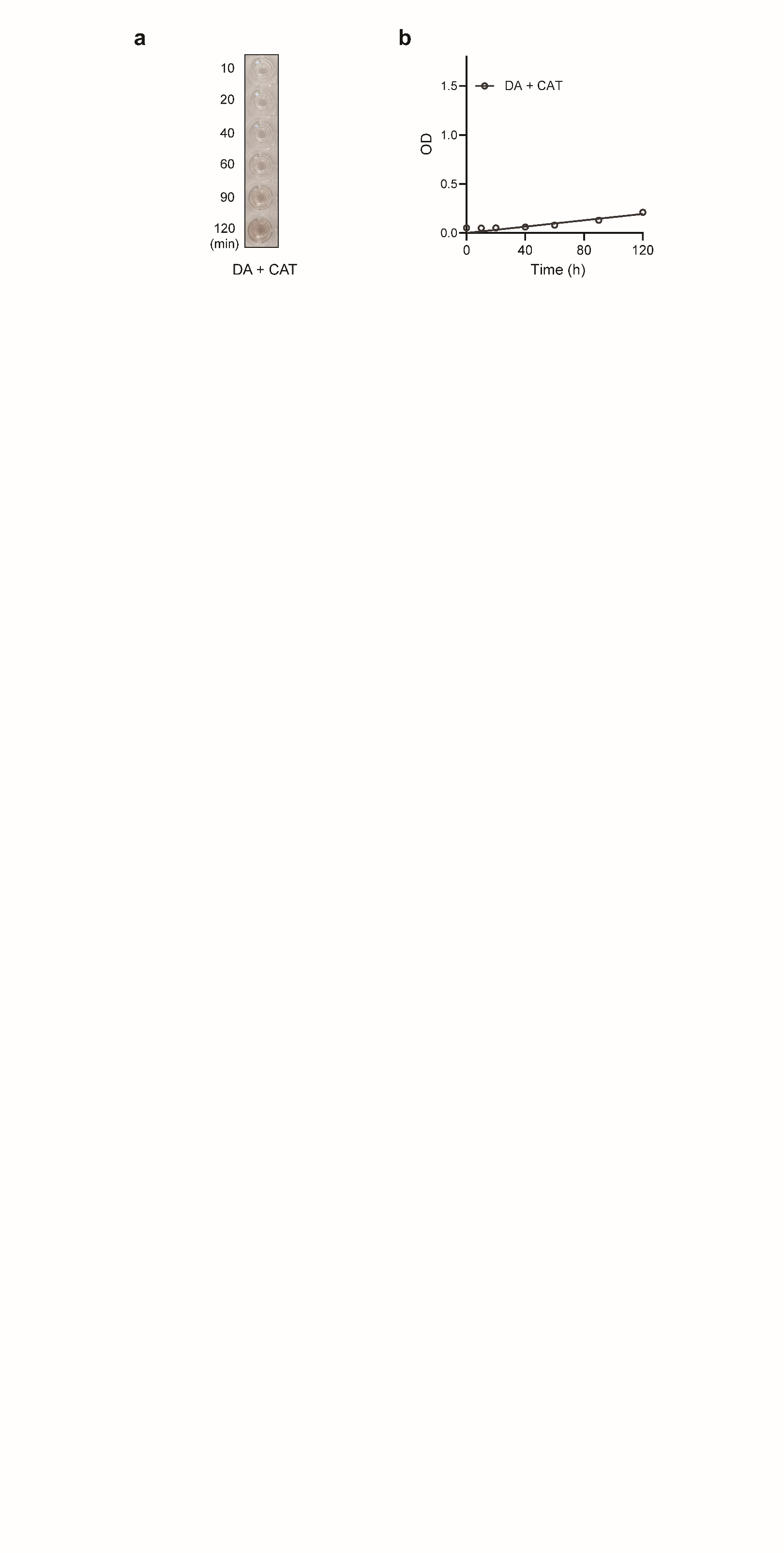


**Figure S2**. a) Visual results of PDA polymerization at various time points (DA is colorless and PDA is dark brown). b) OD value of the samples in (a) at 700 nm (*n* = 3).


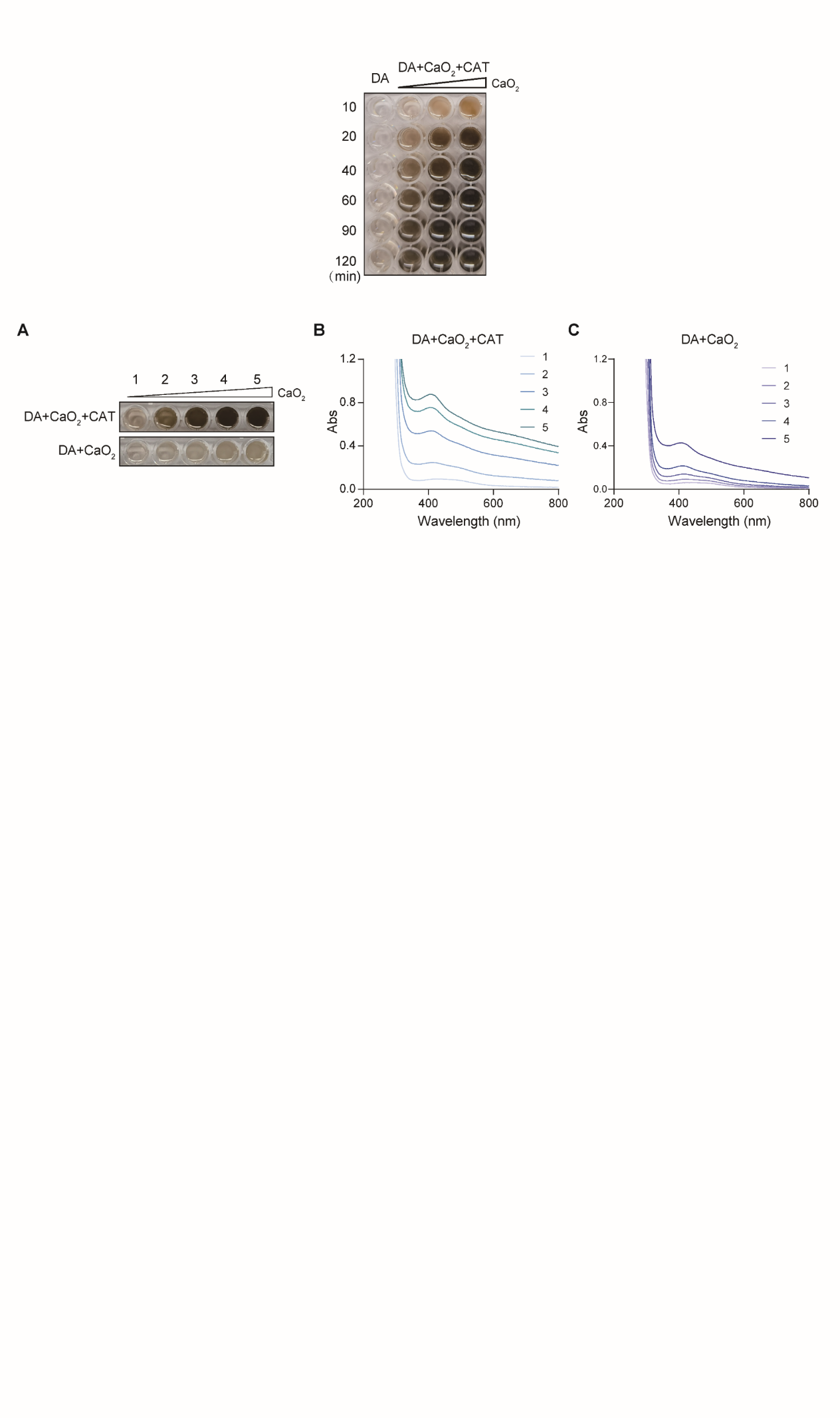


**Figure S3**. PDA polymerization in the presence of CaO_2_ and CAT (The content of CaO_2_ increases gradually).


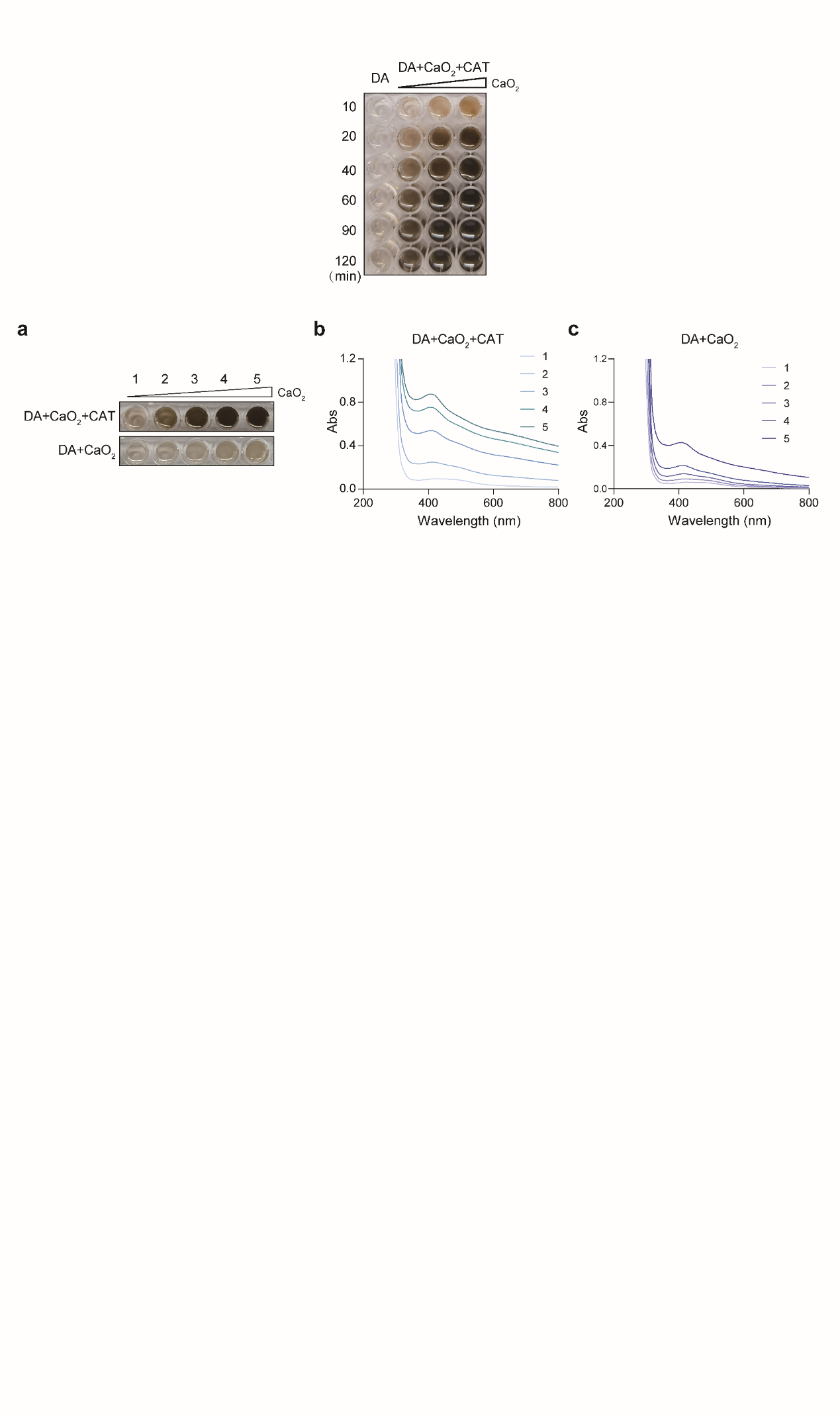


**Figure S4**. Catalysis of CAT. a) Effects of CAT on DA and CaO_2_ responses. b, c) UV-vis spectra of PDA under the condition in (a) (The content of CaO_2_ increases gradually from 1 to 5).


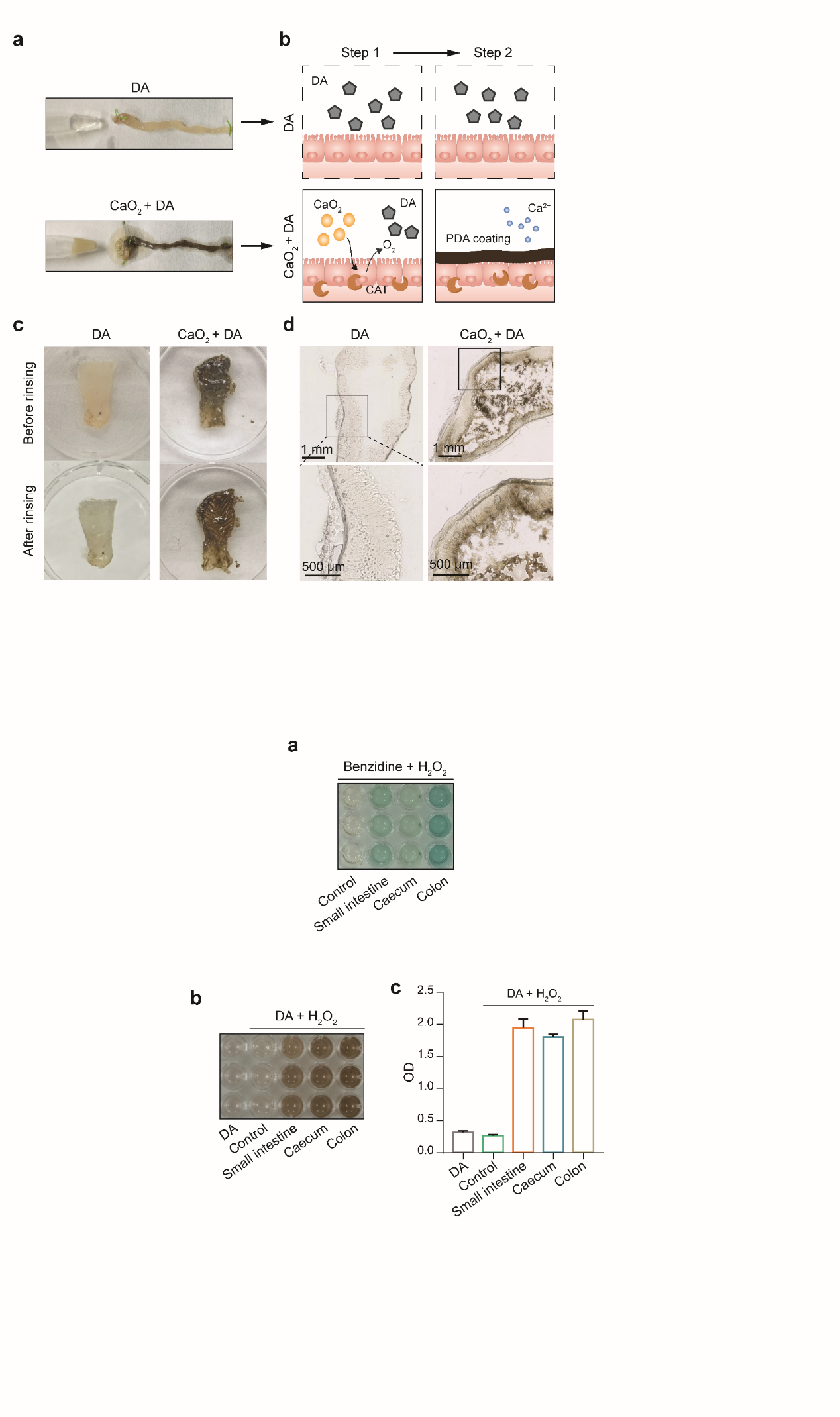


**Figure S5**. Expression of CAT in intestinal tissue was detected by Benzidine and H_2_O_2_, and the colon has the highest levels of CAT (the blue color of benzidine indicates the level of CAT).


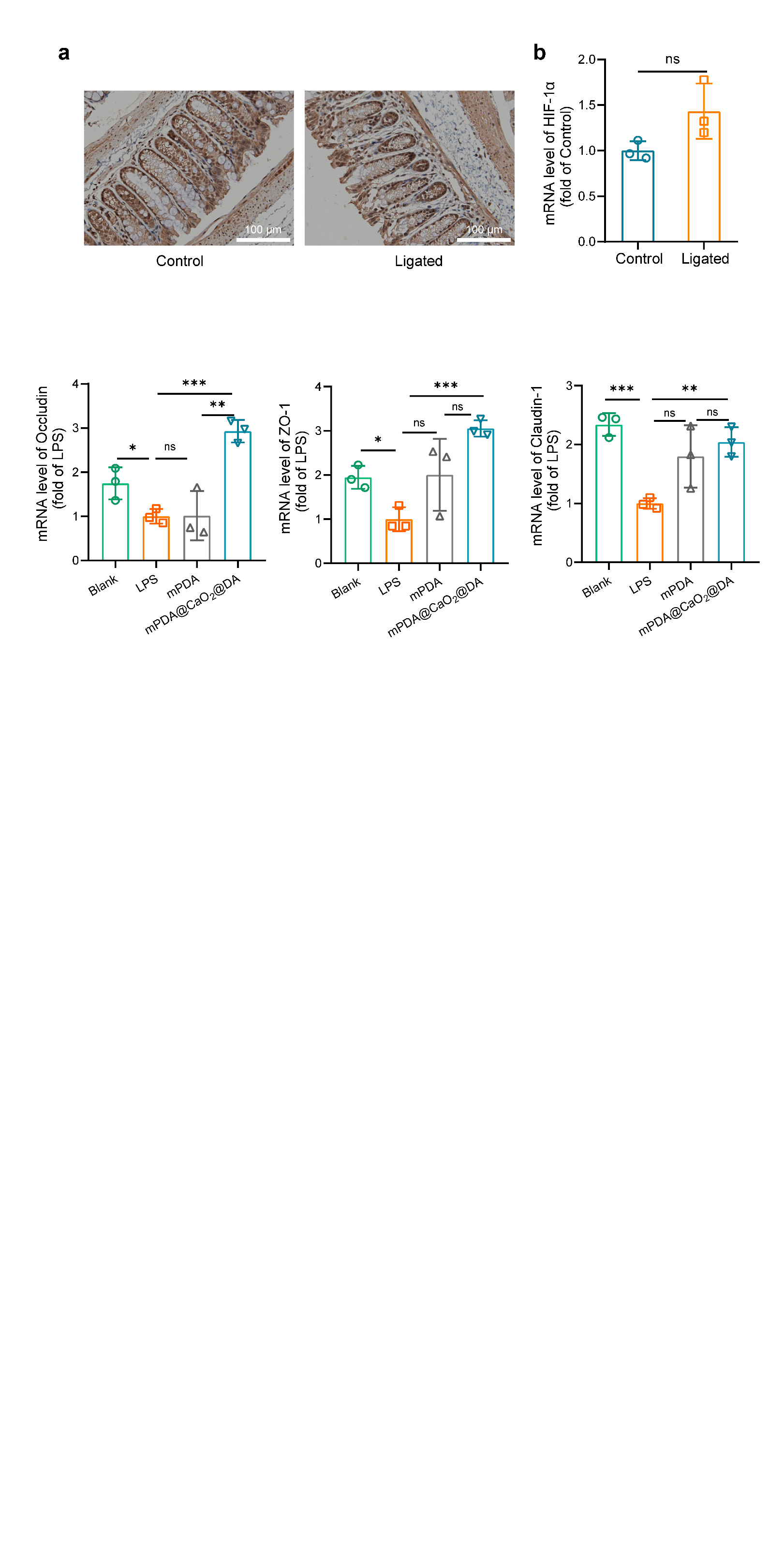


**Figure S6.** a) Immunohistochemical staining of HIF-1α in control and ligated colon. b) The mRNA level of HIF-1α in control and ligated colon (*n* = 3). Results were expressed as mean ± SD. ns represented not significant.


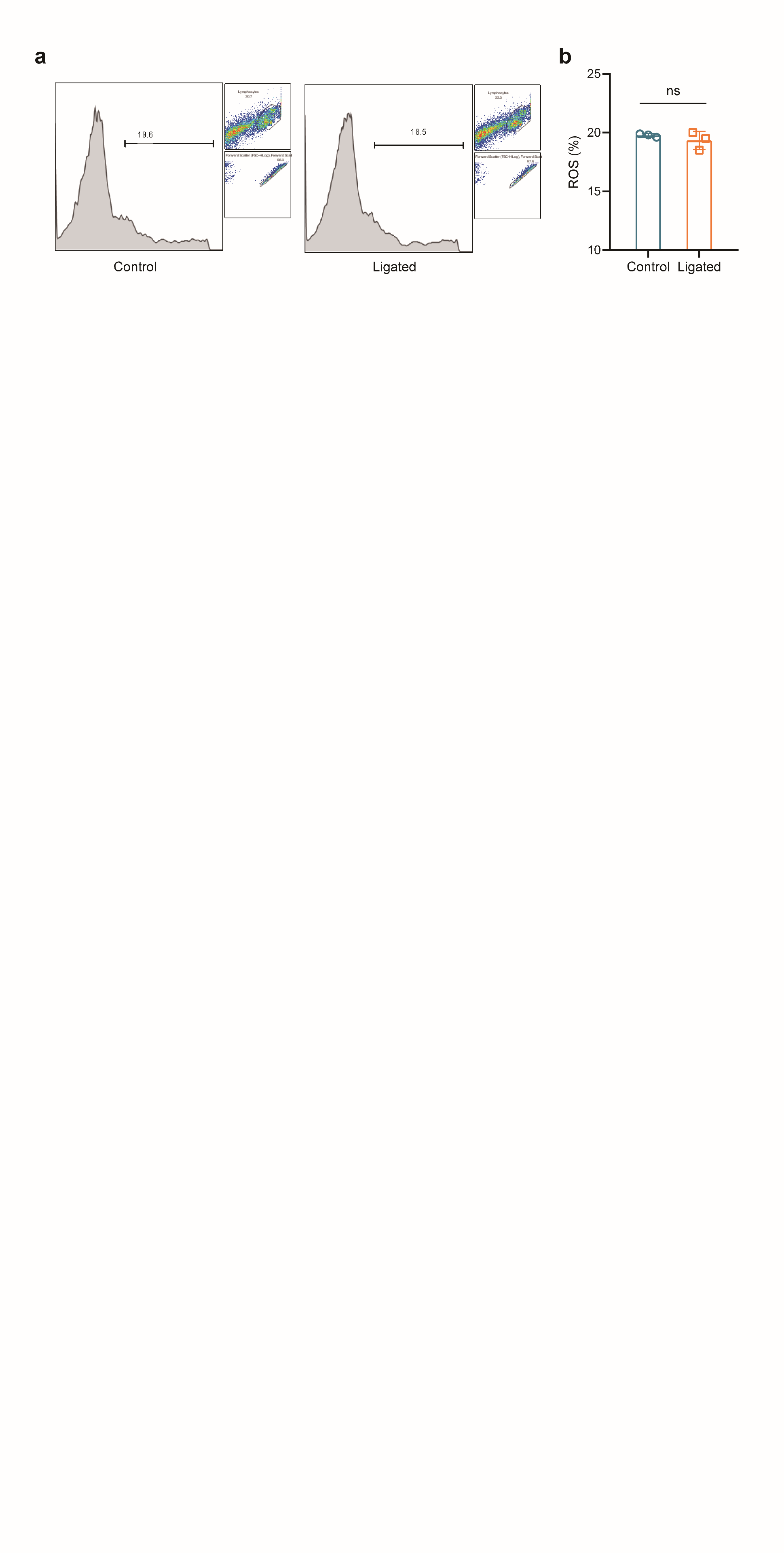
**Figure S7.** a) The flow cytometry analysis of ROS level in control and ligated colon. b) Quantitative results of ROS level in (a) (*n* = 3). Results were expressed as mean ± SD. ns represented not significant.


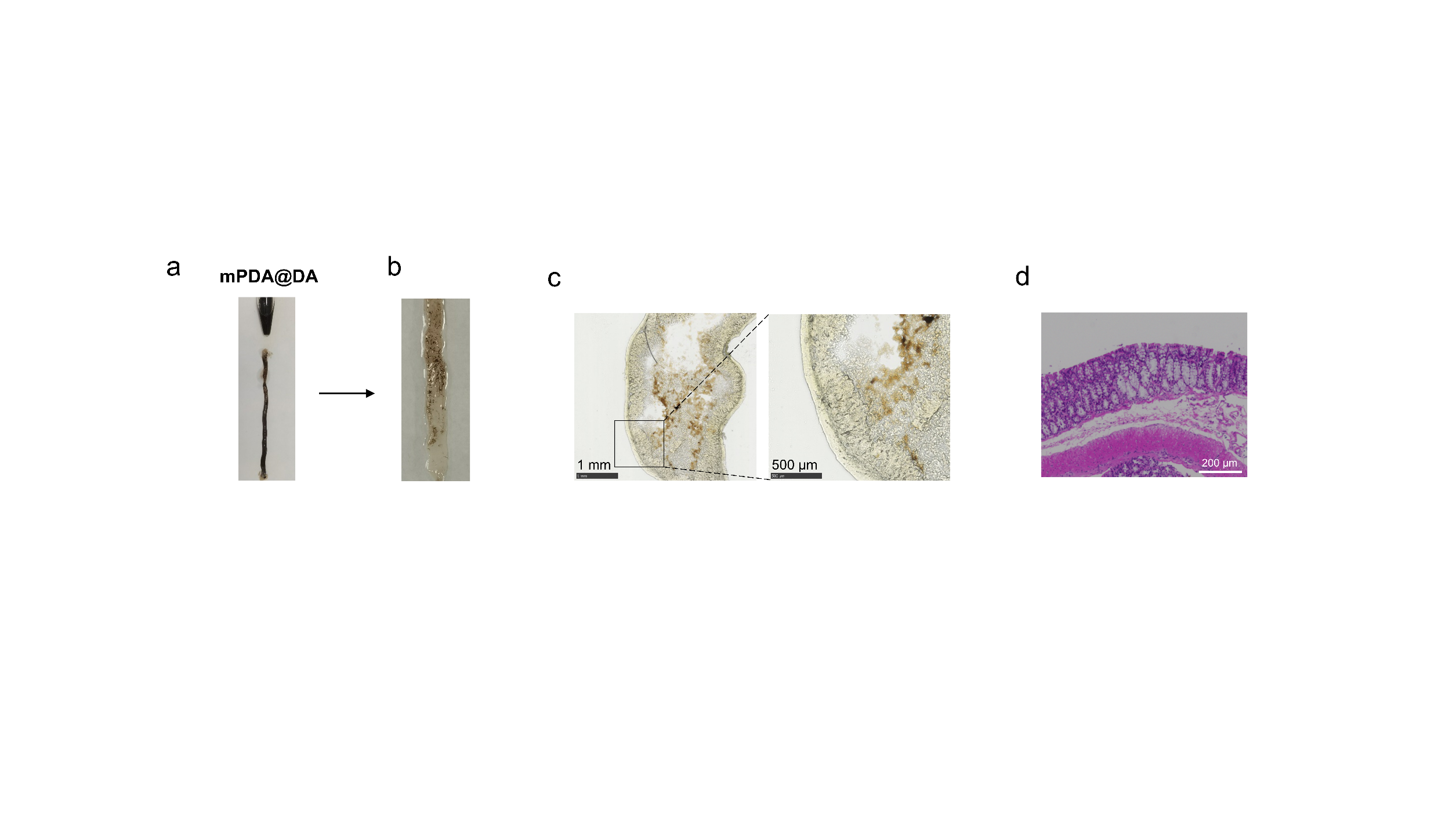


**Figure S8**. a) The colon was incubated with mPDA@DA. b) Image of the intestine. c) Microscopic view of the the epithelium. d) H&E staining of the colon.


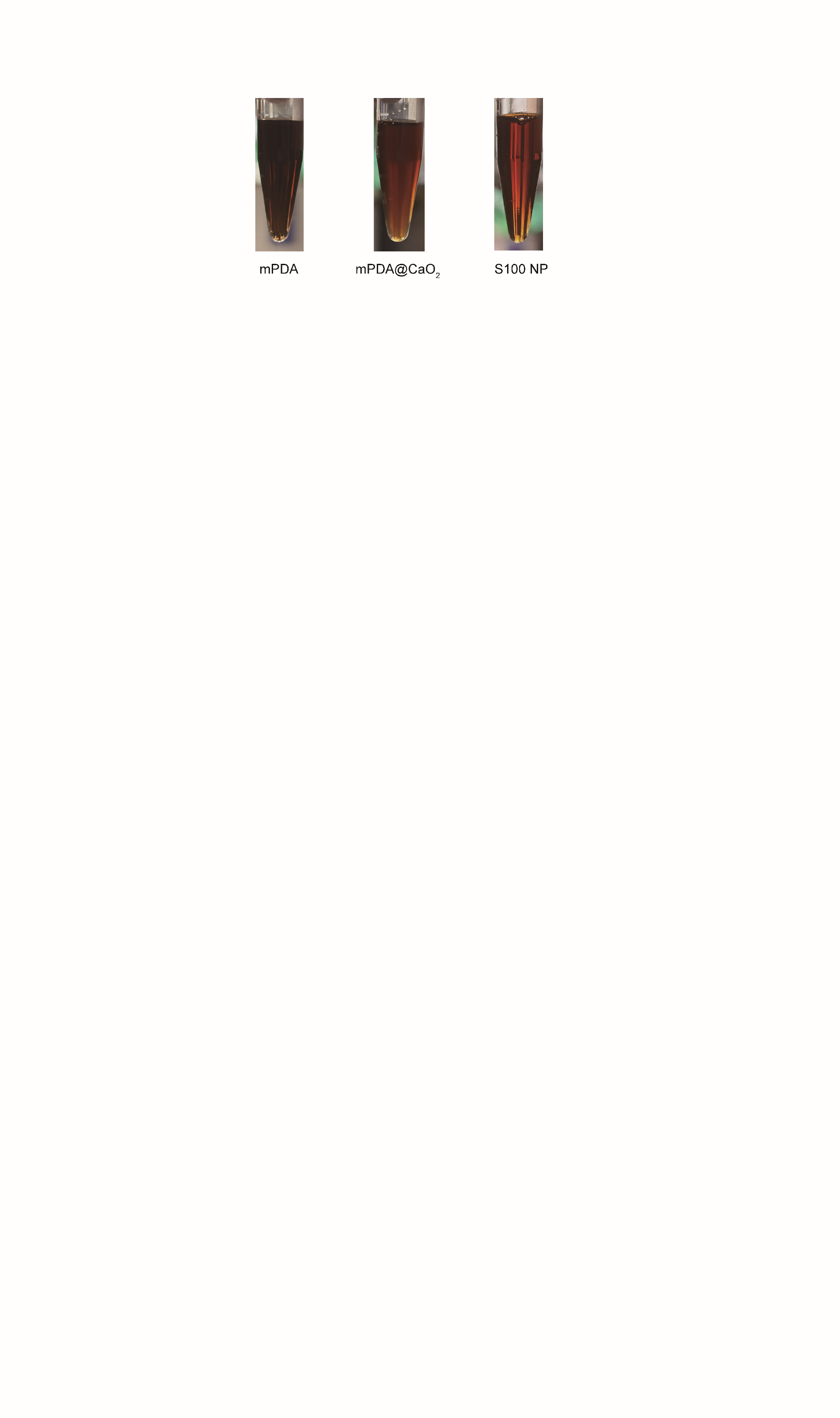


**Figure S9**. Images of the mPDA, mPDA@CaO_2_, and S100 NP.


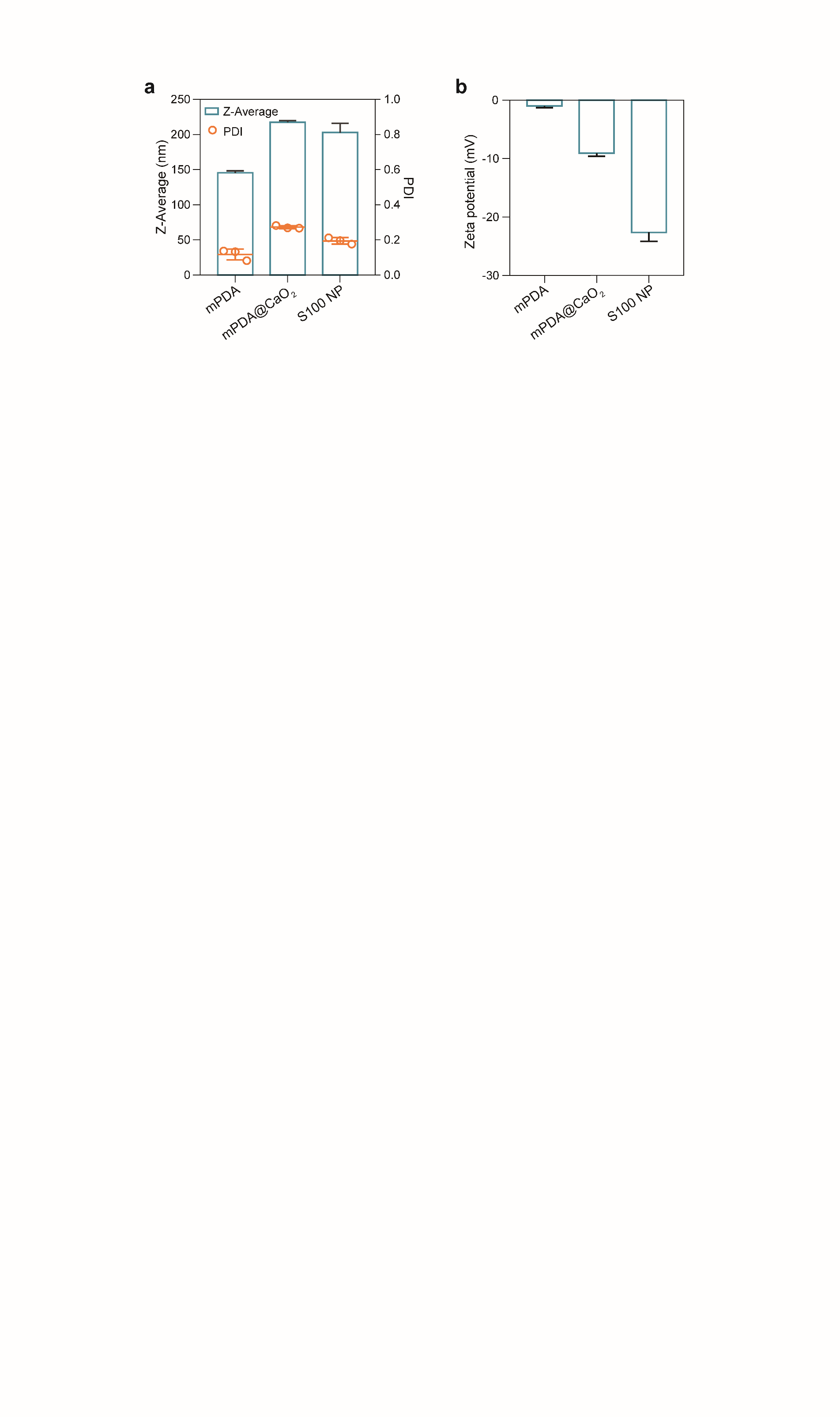
**Figure S10**. Characterization of the nanoparticles. a) Size and PDI. b) Zeta potential (*n* = 3). Results were expressed as mean ± SD.


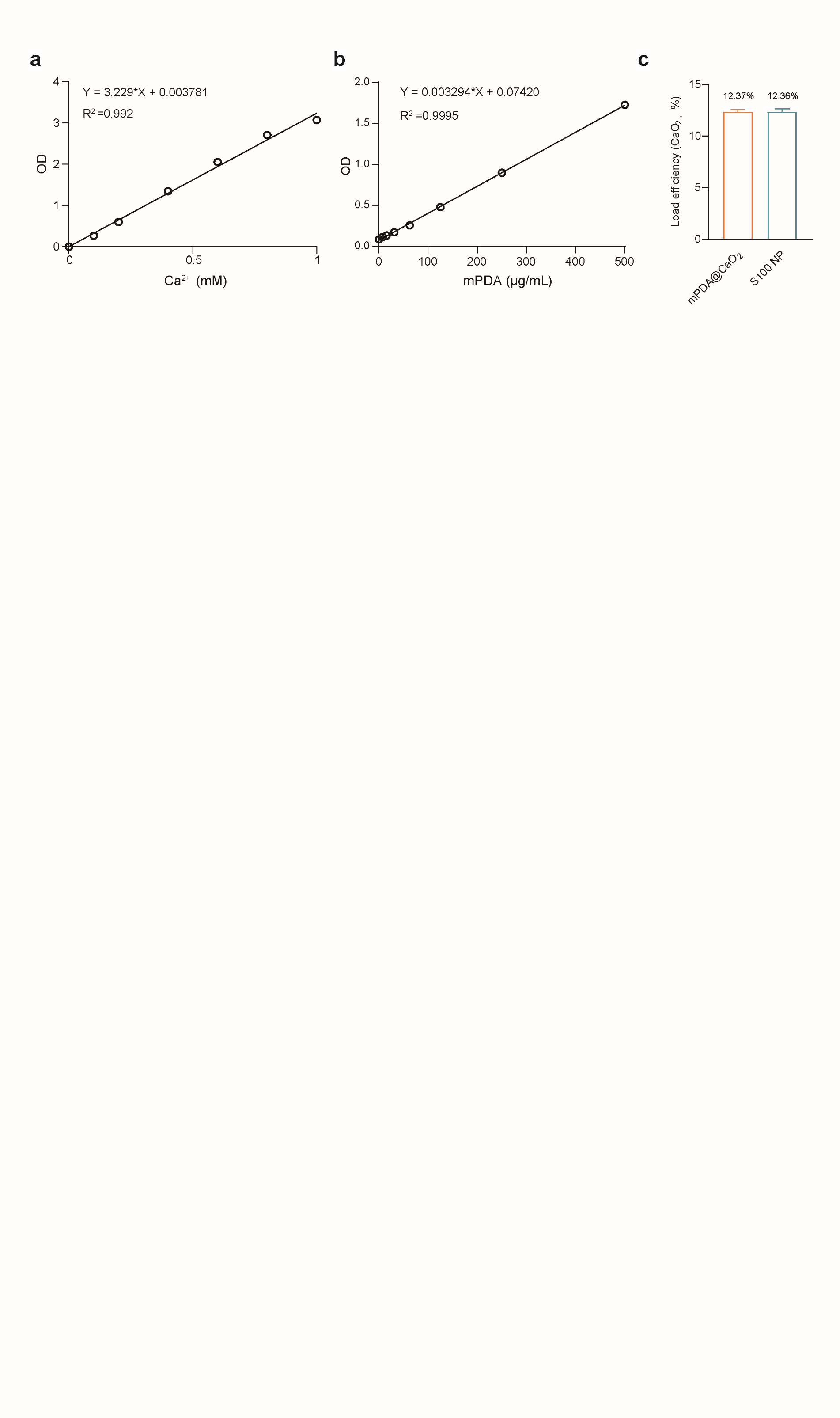


**Figure S11**. The concentration standard curve of a) Ca^2+^ and b) mPDA.


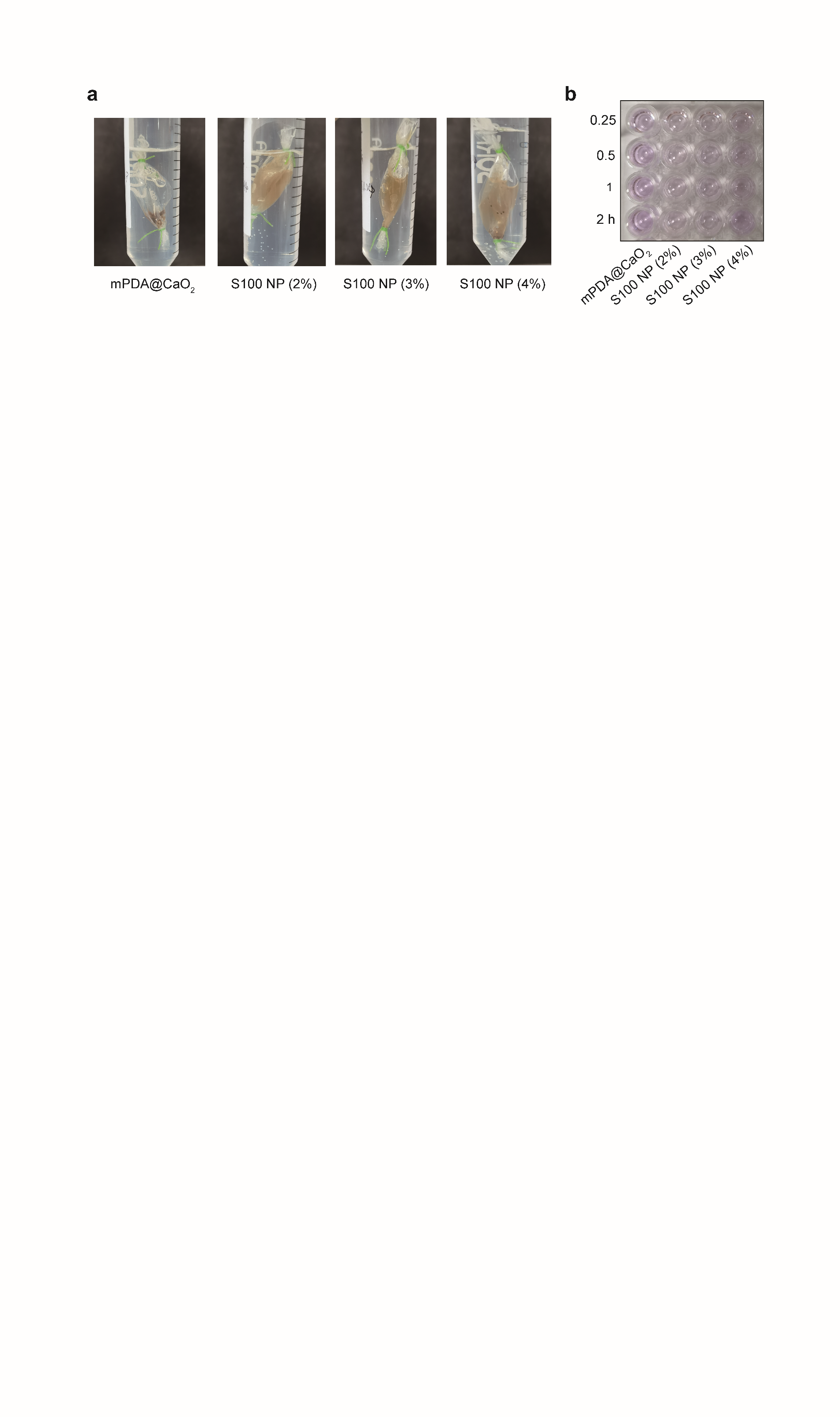


**Figure S12**. The S100 layer protects S100 NP in an acid environment. a) Release of Ca^2+^ from nanoparticles in an acidic environment (pH 2). b) Image of Ca^2+^ detection (The purple color indicates Ca^2+^).


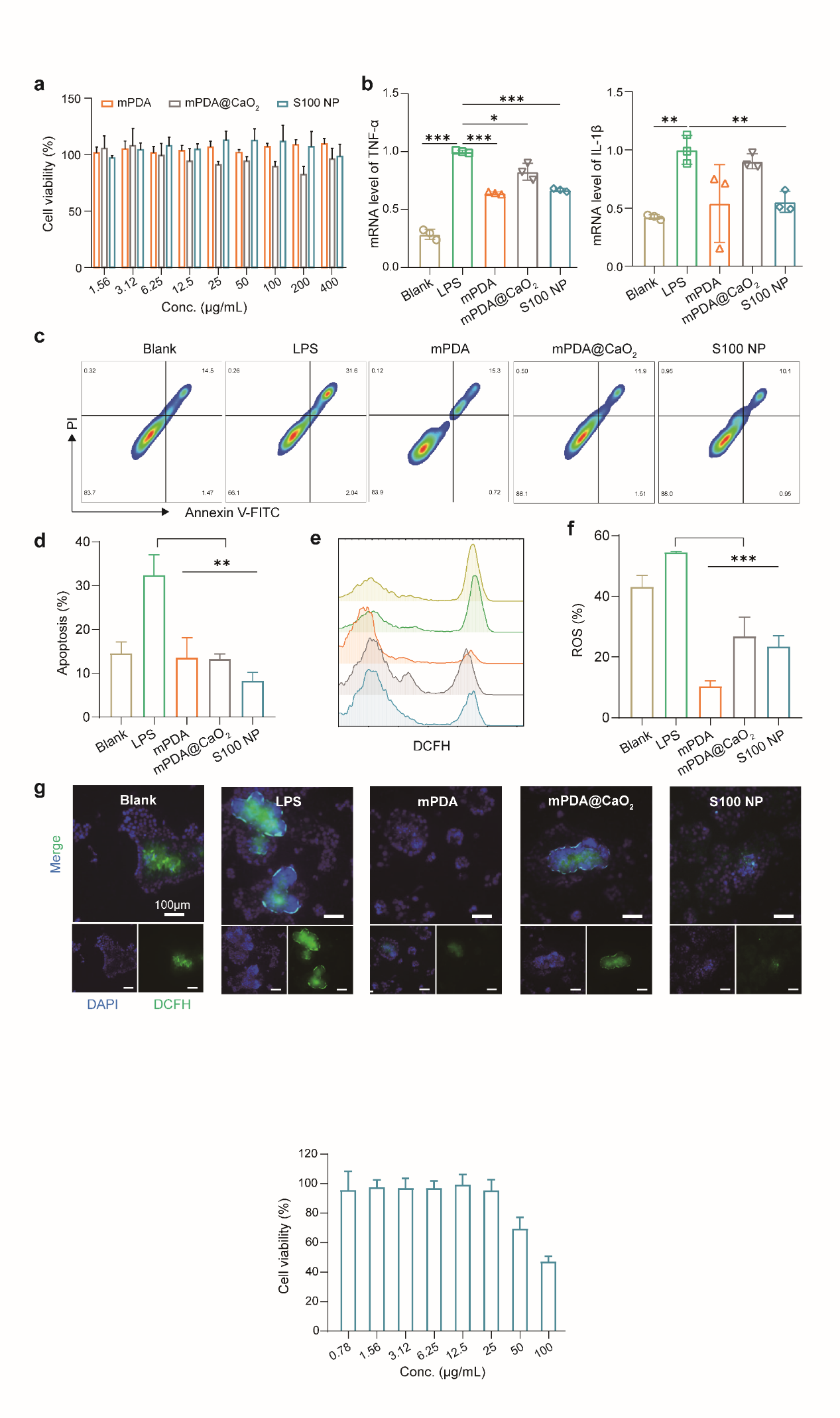
**Figure S13**. Cell viability of the LPS-treated Caco2 (*n* = 3). Results were expressed as mean ± SD.

**Figure S1**
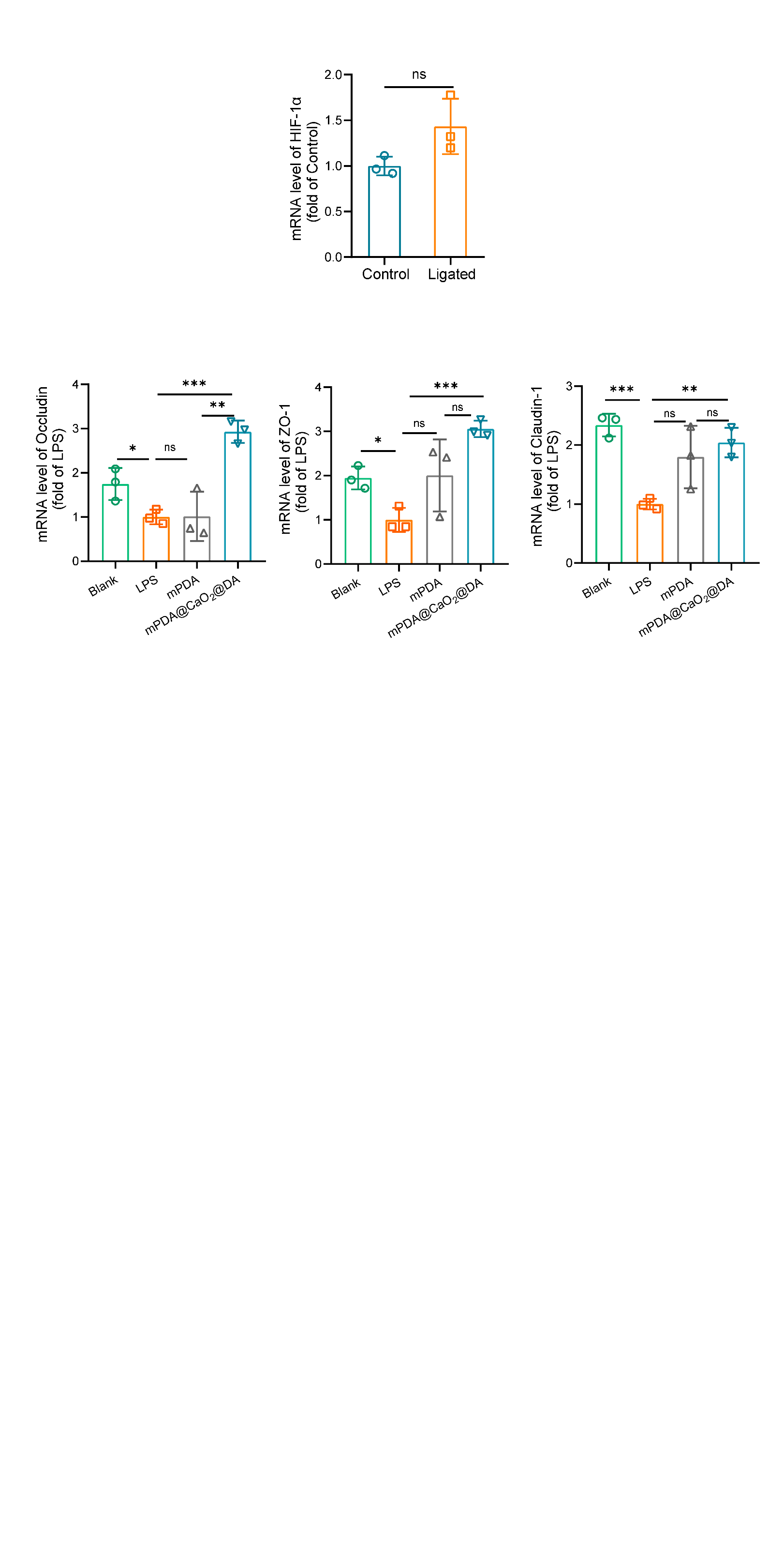
**4.** The mRNA level of Occludin, ZO-1, and Claudin-1 (*n* = 3). Results were expressed as mean ± SD. **p* <0.05, ***p* <0.01, ****p* < 0.001. ns represented not significant.


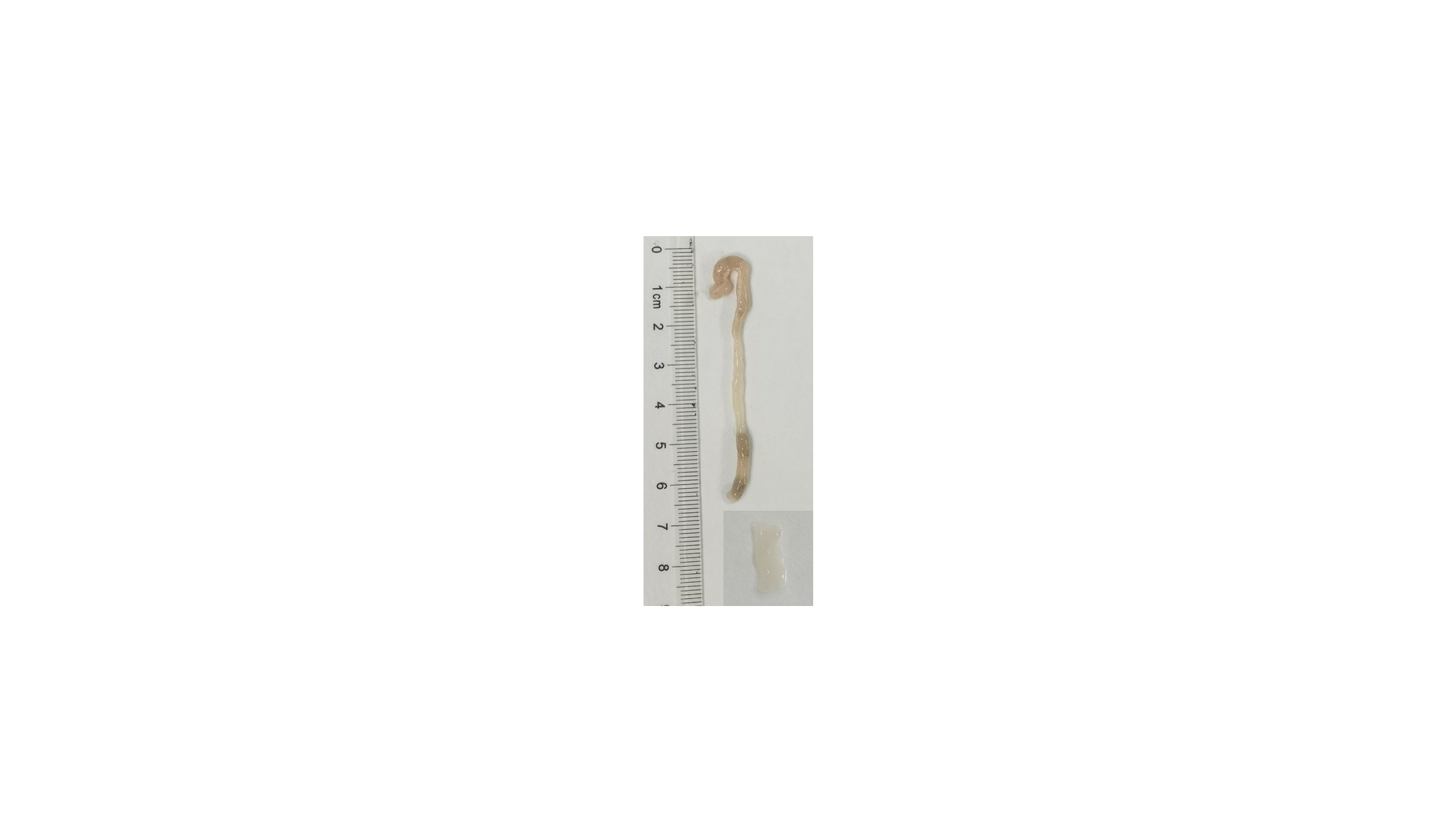


**Figure S15**. The colon of DSS-induced colitis mice after oral administration of DA.


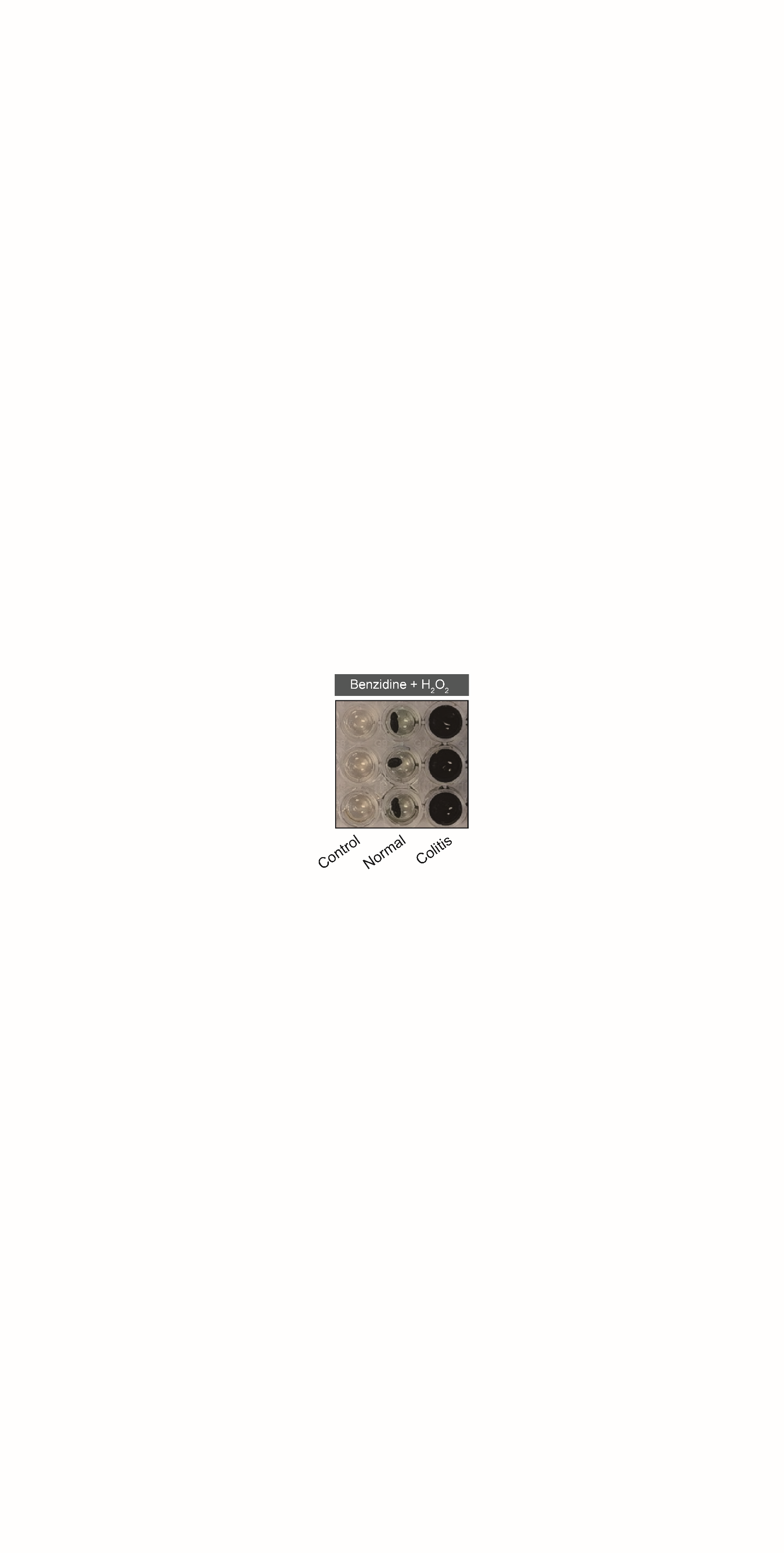
**Figure S16**. Expression of CAT in the intestine of normal mice and TNBS-induced colitis mice was detected by Benzidine and H_2_O_2_.


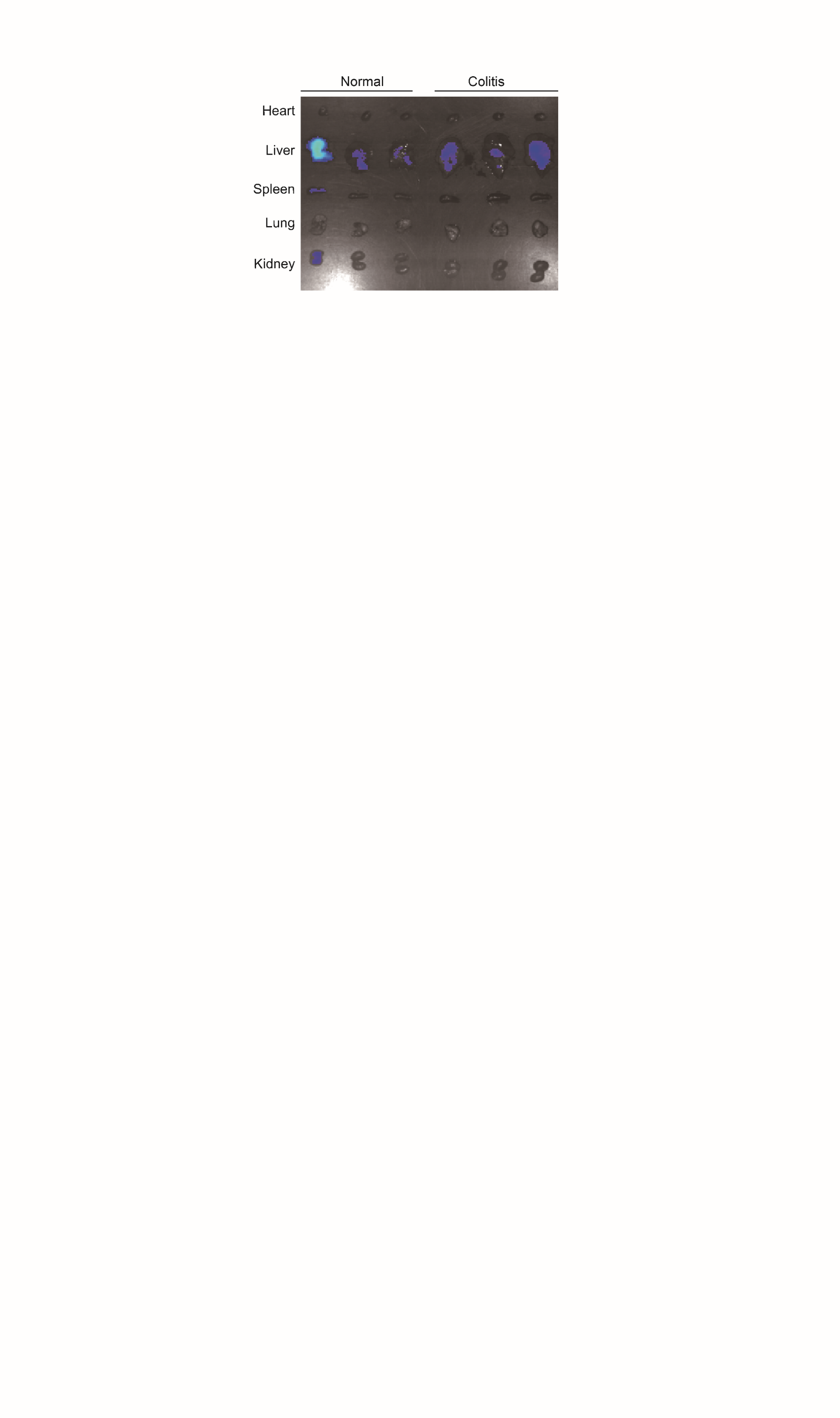


**Figure S17**. Ex vivo radiant efficiency of the major organs in normal and colitis mice.

**
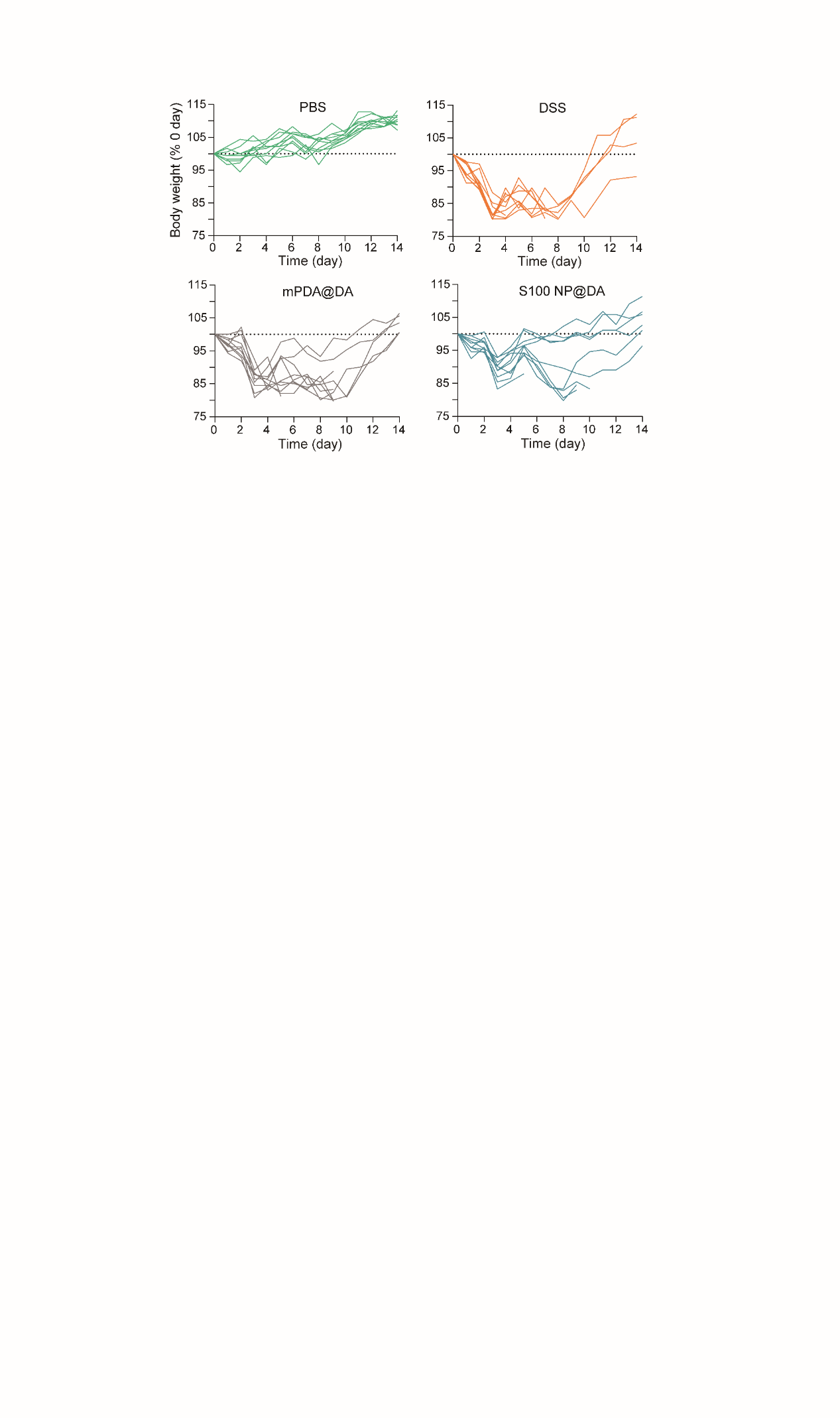
**

**Figure S18**. Individual body weight of PBS, DSS, mPDA@DA, and S100 NP@DA groups.


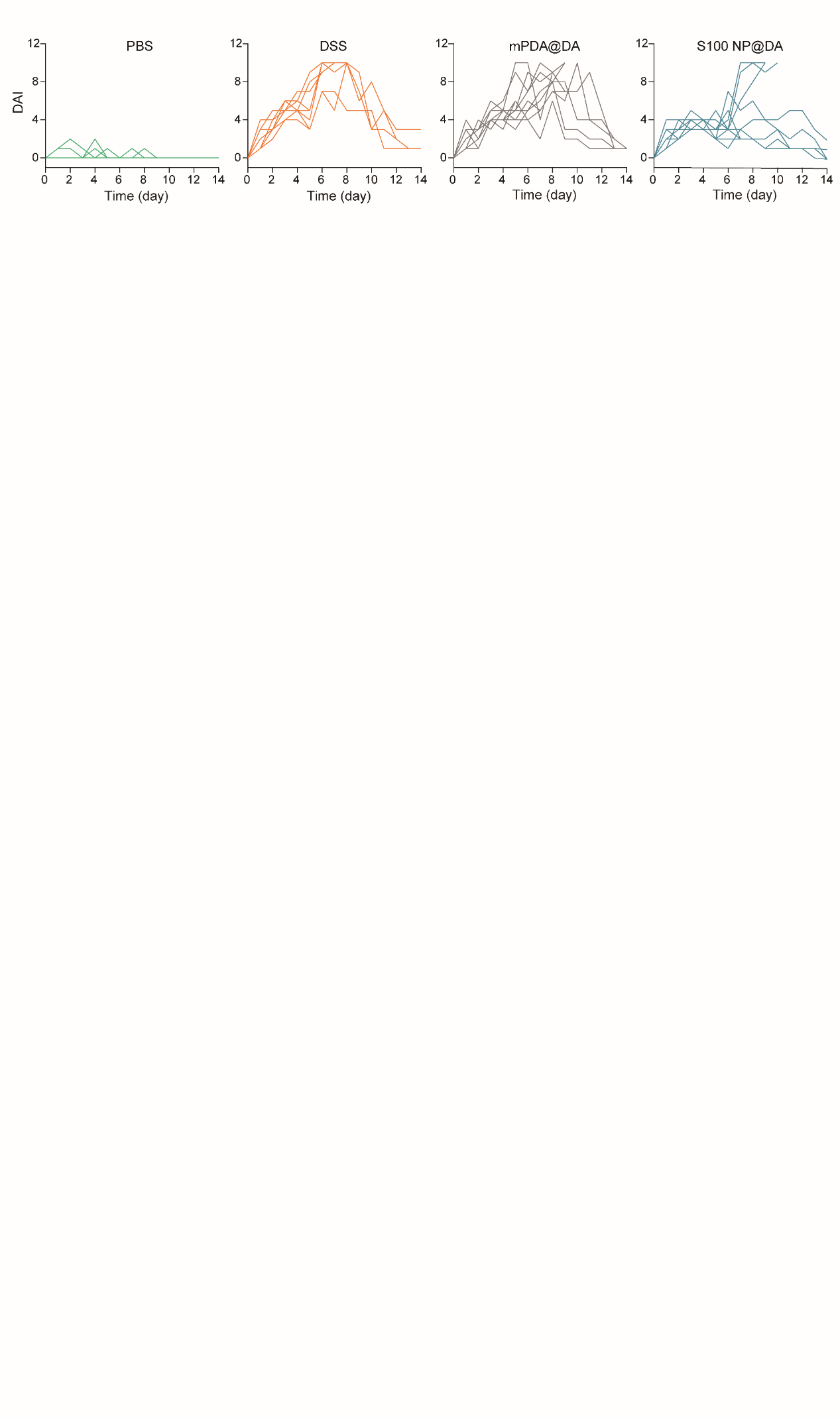


**Figure S19**. Individual DAI of PBS, DSS, mPDA@DA, and S100 NP@DA groups.


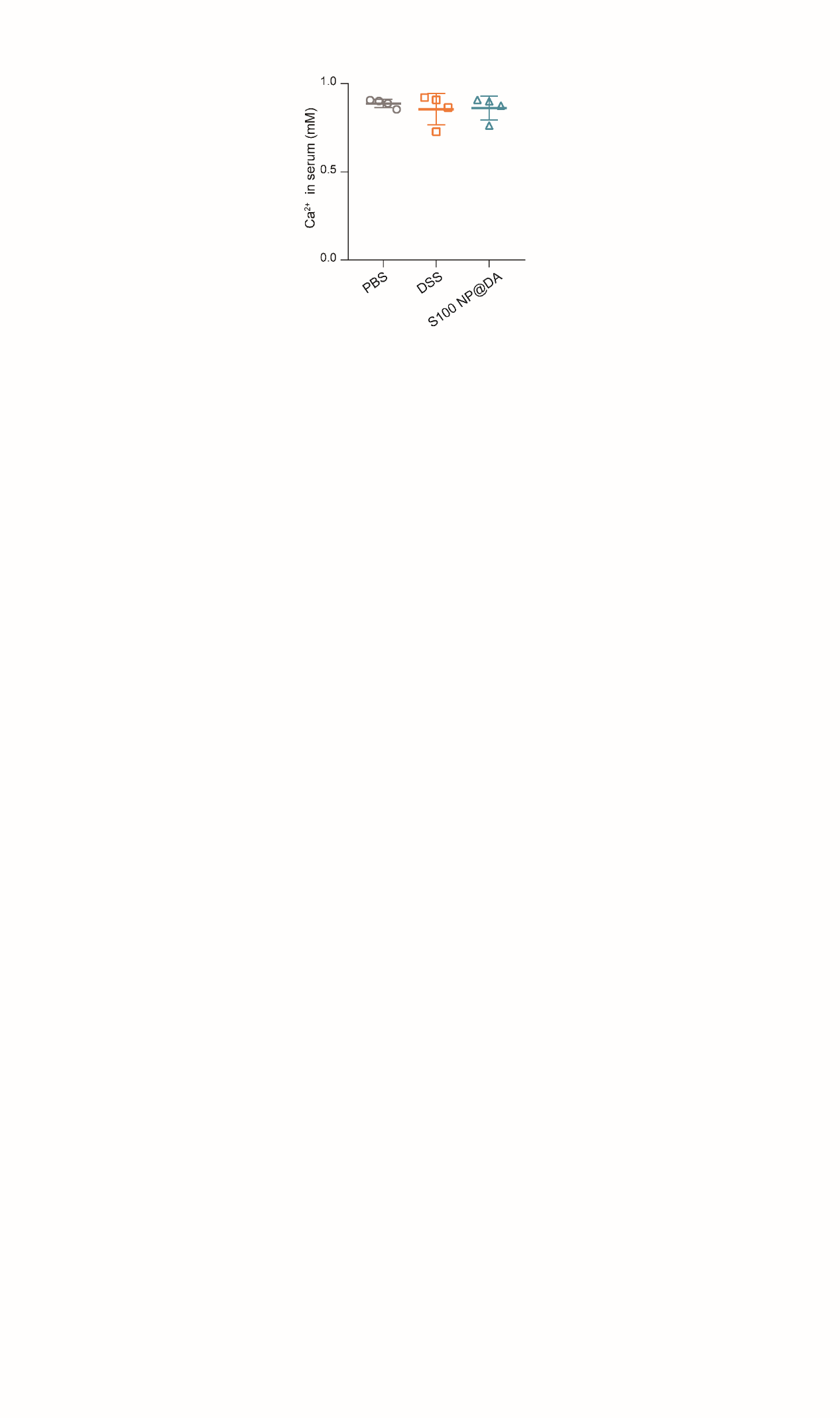
**Figure S20**. Ca^2+^ concentration in serum (*n* = 3). Results were expressed as mean ± SD.


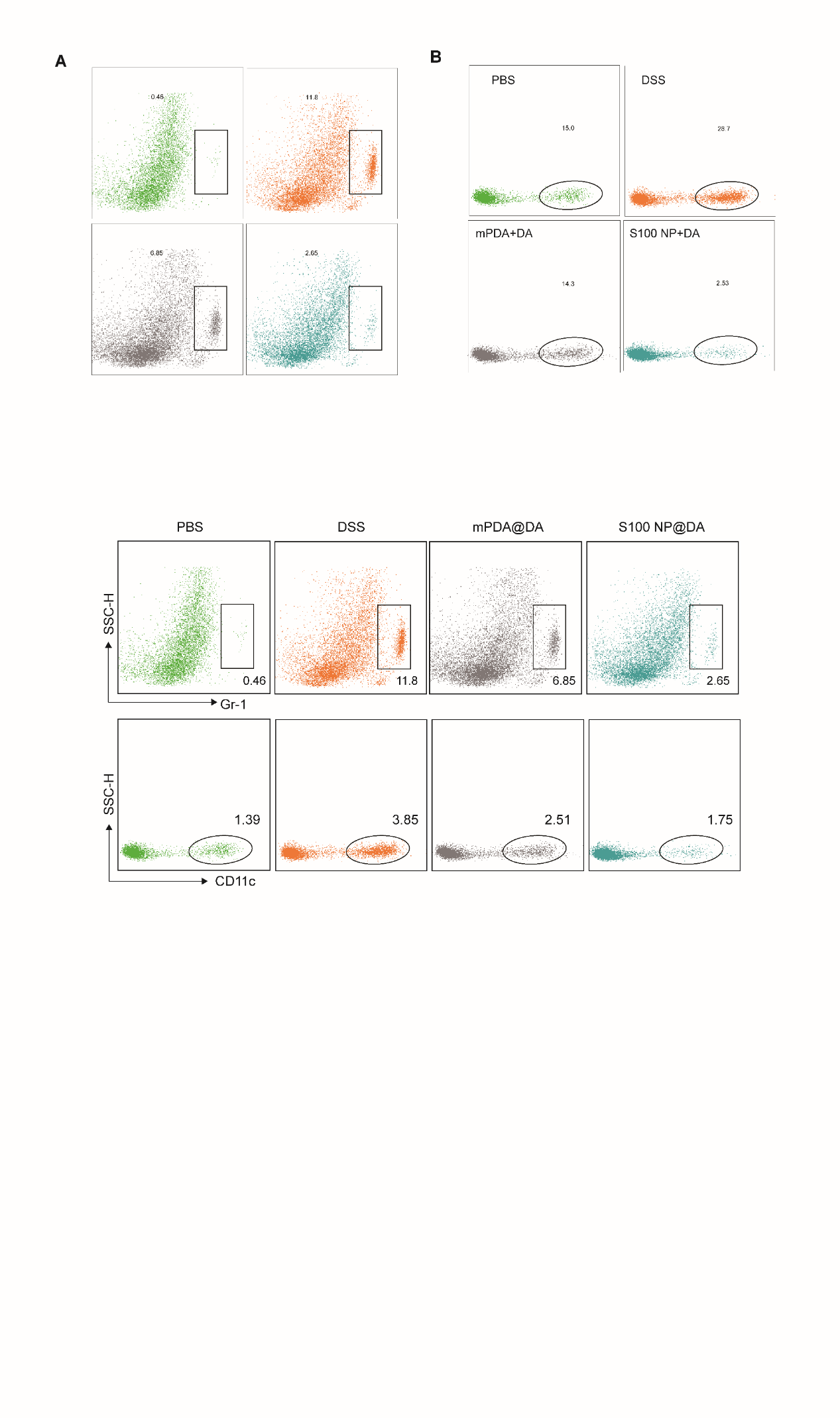


**Figure S21**. Flow cytometry analysis of neutrophils (Gr-1^+^) and dendritic cells (CD11c^+^).


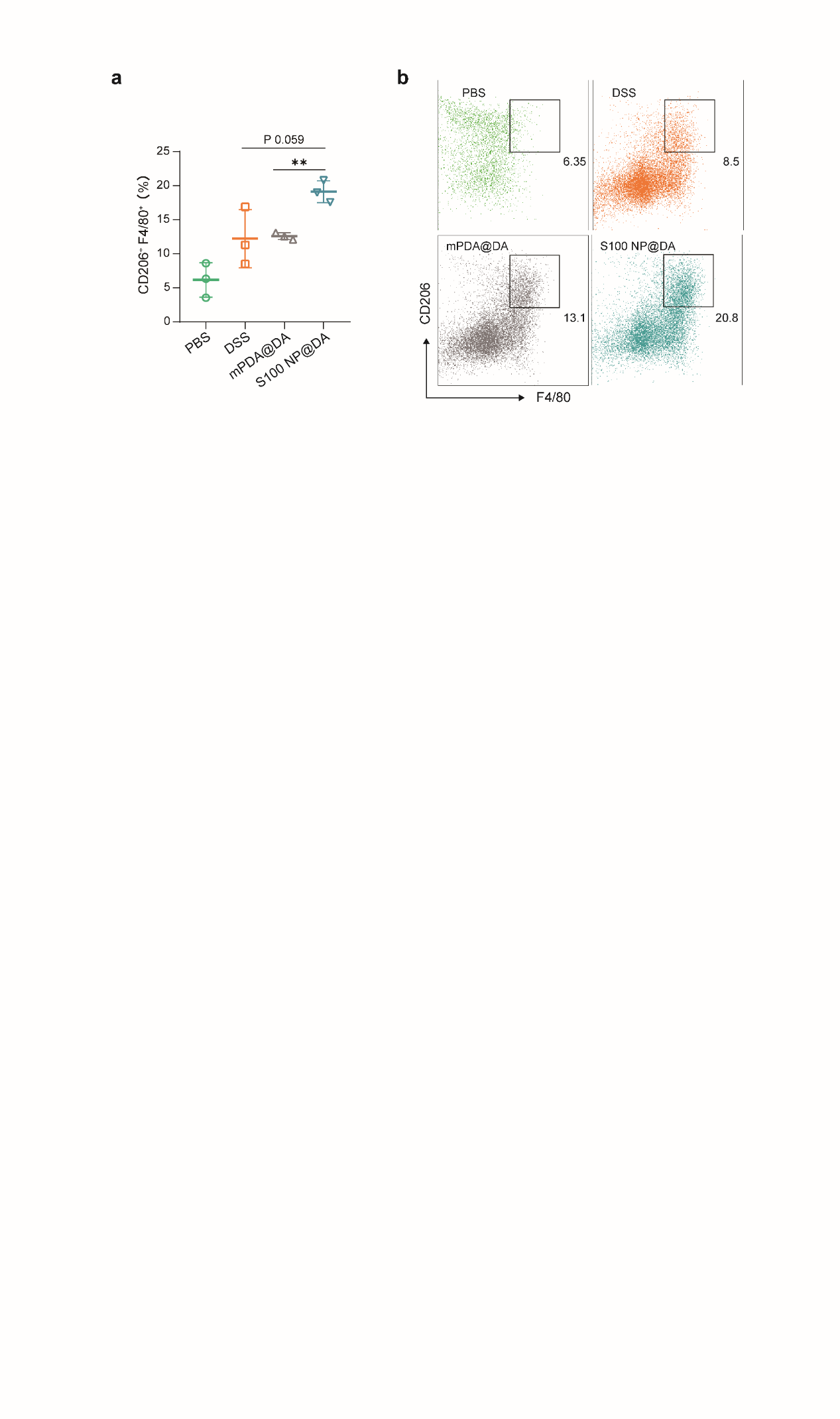


**Figure S22**. a) The population (*n* = 3) and b) flow cytometry analysis of M2 macrophages. Results were expressed as mean ± SD.


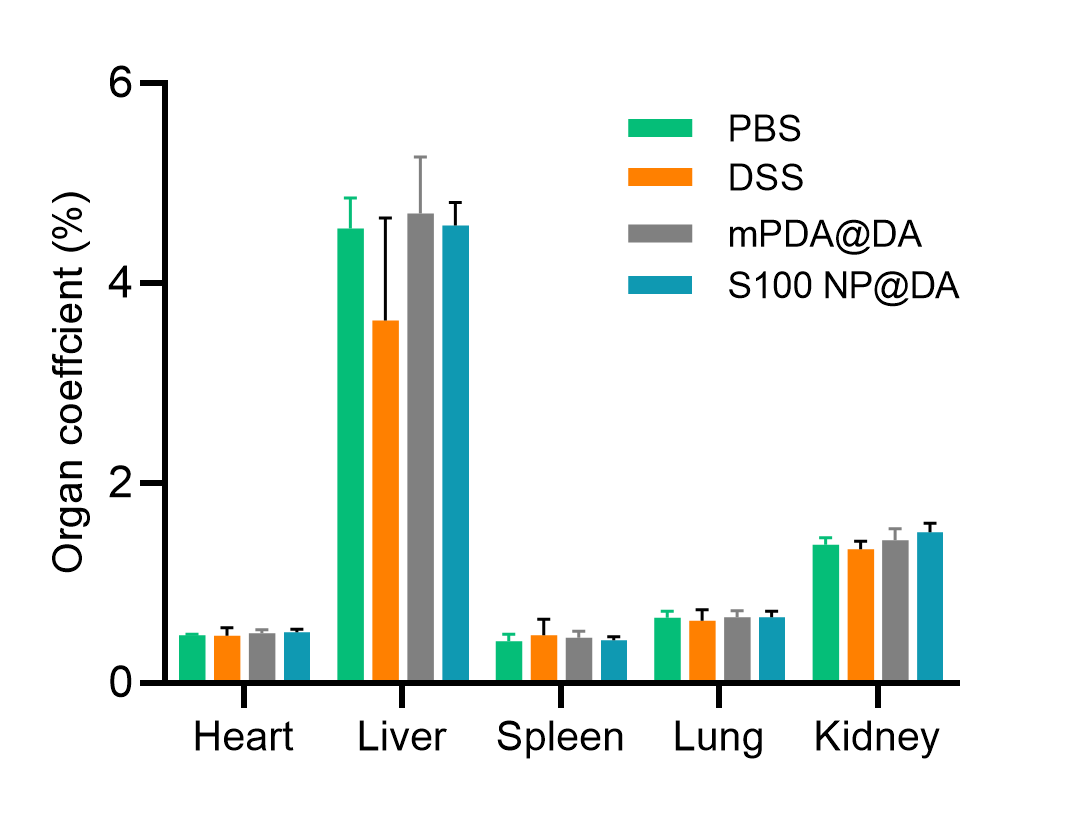


**Figure S23**. Organ coefficients (*n* = 4–6, There was no significant difference among the groups).


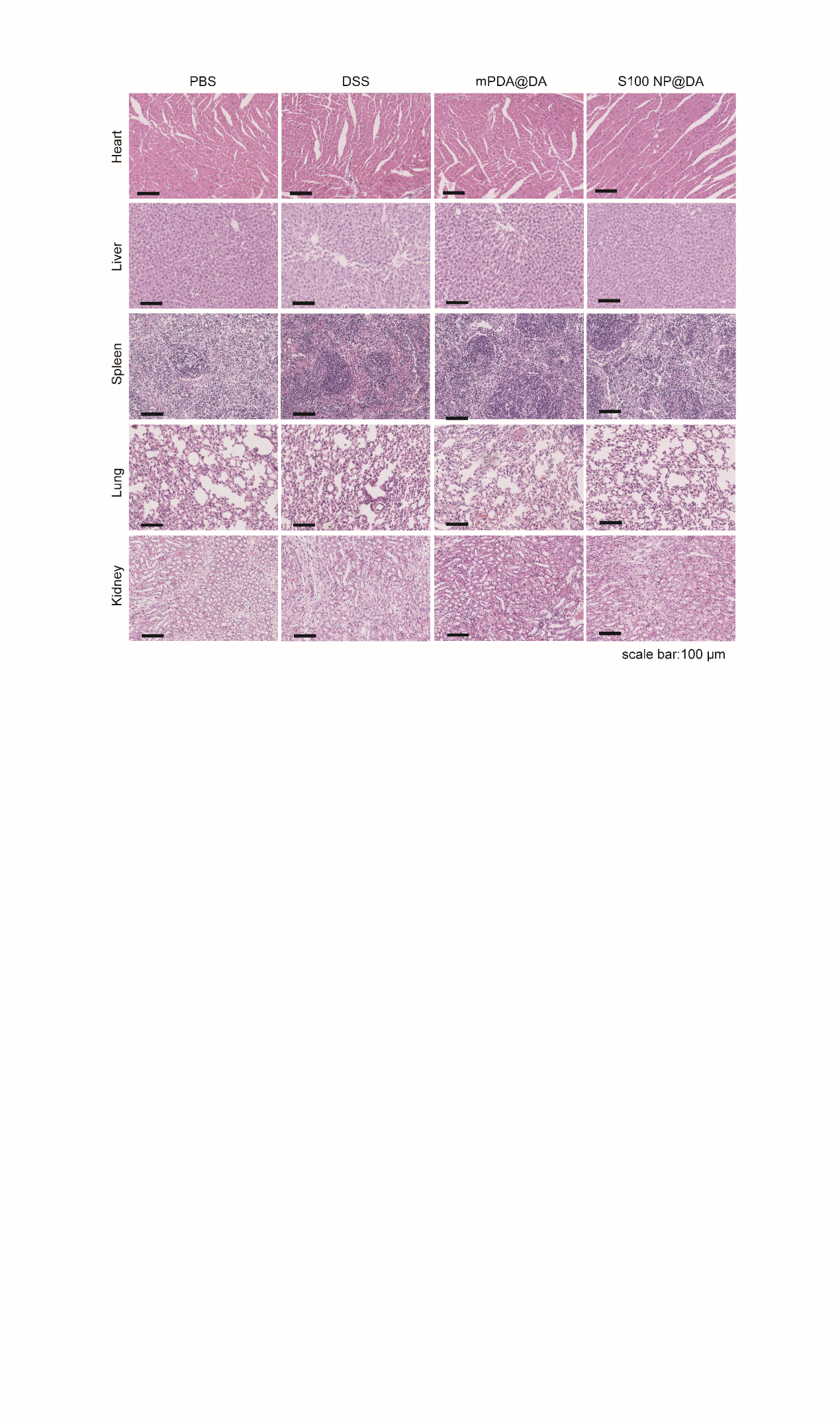


**Figure S24**. The histological examination of the major organs (scale bar: 100 μm). There were no obvious pathological changes.
